# Esketamine/Ketamine: Dual‐Action Mechanisms and Clinical Prospects beyond Anesthesia in Psychiatry, Immunology, and Oncology

**DOI:** 10.1002/advs.202516024

**Published:** 2025-12-14

**Authors:** Yinxin Wang, Jiangang Xu, Xinyu Zhou, Lu Che, Minghan Qiu, Yang Liu, Ming Gao, Zhen Yang

**Affiliations:** ^1^ School of Medicine Nankai University Tianjin 300071 China; ^2^ The Institute of Translational Medicine Tianjin Cancer Institute of Integrative Traditional Chinese and Western Medicine Tianjin Union Medical Center The First Affiliated Hospital of Nankai University Nankai University Tianjin 300121 China; ^3^ Department of Anesthesiology Tianjin Union Medical Center The First Affiliated Hospital of Nankai University Nankai University Tianjin 300121 China; ^4^ Department of Anesthesiology Tianjin Central Hospital of Gynaecology Obsterics Nankai University Tianjin 300071 China; ^5^ Department of Anesthesiology Tianjin First Central Hospital Tianjin 300110 China; ^6^ Key Laboratory of Functional Polymer Materials of Ministry of Education College of Chemistry State Key Laboratory of Medicinal Chemical Biology Frontiers Science Center for New Organic Matter Nankai University Tianjin 300071 China

**Keywords:** analgesia, antidepressant, antitumor, esketamine, immune‐inflammatory, ketamine

## Abstract

Esketamine and ketamine are perioperative analgesics and anesthetics that have been widely adopted in clinical practice in Europe and the United States. However, their application in China remains in the early stages of exploration and implementation. Esketamine and ketamine exert their effects through multiple receptors and pathways, but primarily act through non‐competitive antagonism of the N‐methyl‐D‐aspartate receptor (NMDAR). However, due to their potential side effects and addictive properties, they are subject to certain regulatory controls and are not readily included in routine pharmaceutical use. This review meticulously and systematically synthesizes the latest research progress on Esketamine and ketamine. Their classical applications, including analgesia, sedation, and anesthesia, as well as antidepressant, anti‐tumor, and anti‐inflammatory effects, are comprehensively covered. By integrating findings from both preclinical and clinical investigations and taking into full account potential limitations, this review aims to provide profound insights that can effectively inform the clinical utilization and future research directions of ketamine and esketamine as versatile therapeutic agents.

## Graphical abstract

Esketamine and ketamine are widely used for perioperative analgesia and anesthesia. Despite their established roles in analgesia, sedation, and anesthesia, as well as emerging antidepressant, anti‐tumor, and anti‐inflammatory effects, their clinical use is limited due to side effects and addiction potential. This review synthesizes preclinical and clinical evidence to guide future research and safe clinical practice.

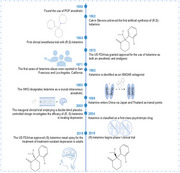

## 1 Introduction

Ketamine, chemically designated as C13H16ClNO, and also recognized by its systematic name 2‐(2‐chlorophenyl)‐2‐(methylamino) cyclohexanone, is a potent agent within the phencyclidine (PCP) class of pharmaceuticals. This compound, first crafted by the distinguished Professor Calvin Stevens in the year 1962, was subsequently sanctioned for use as an anesthetic in clinical settings in 1970. Since its first synthesis, research has shown that ketamine has a variety of effects, such as relieving allergic pain and preventing septic and hypovolemic shock, as well as antidepressant, neuroprotective, anti‐inflammatory, and antitumor effects.^[^ ^1^ ^]^ It can be said that ketamine has been used in clinical practice for over six decades, which fully demonstrates its enduring importance and influence.^[^ ^2^, ^3^, ^4^, ^5^ ^]^ However, ketamine also has varying degrees of side effects.^[^ ^6^, ^7^, ^8^, ^9^, ^10^ ^]^ Beyond the recognized clinical side effects, which encompass sedation, dizziness, dissociation, and psychotic symptoms,^[^ ^11^ ^]^ it has a well‐documented history of being misused as a recreational drug. The long‐term recreational use of ketamine without regulation can lead to a series of problems, such as addiction, ulcerative cystitis, hallucinations, and memory disorders.^[^ ^12^, ^13^ ^]^ Given these risk factors, ketamine is strictly controlled when used as a therapeutic drug and must be administered under the supervision of certified medical personnel within the framework of risk assessment and mitigation strategies.

**Figure 1:**
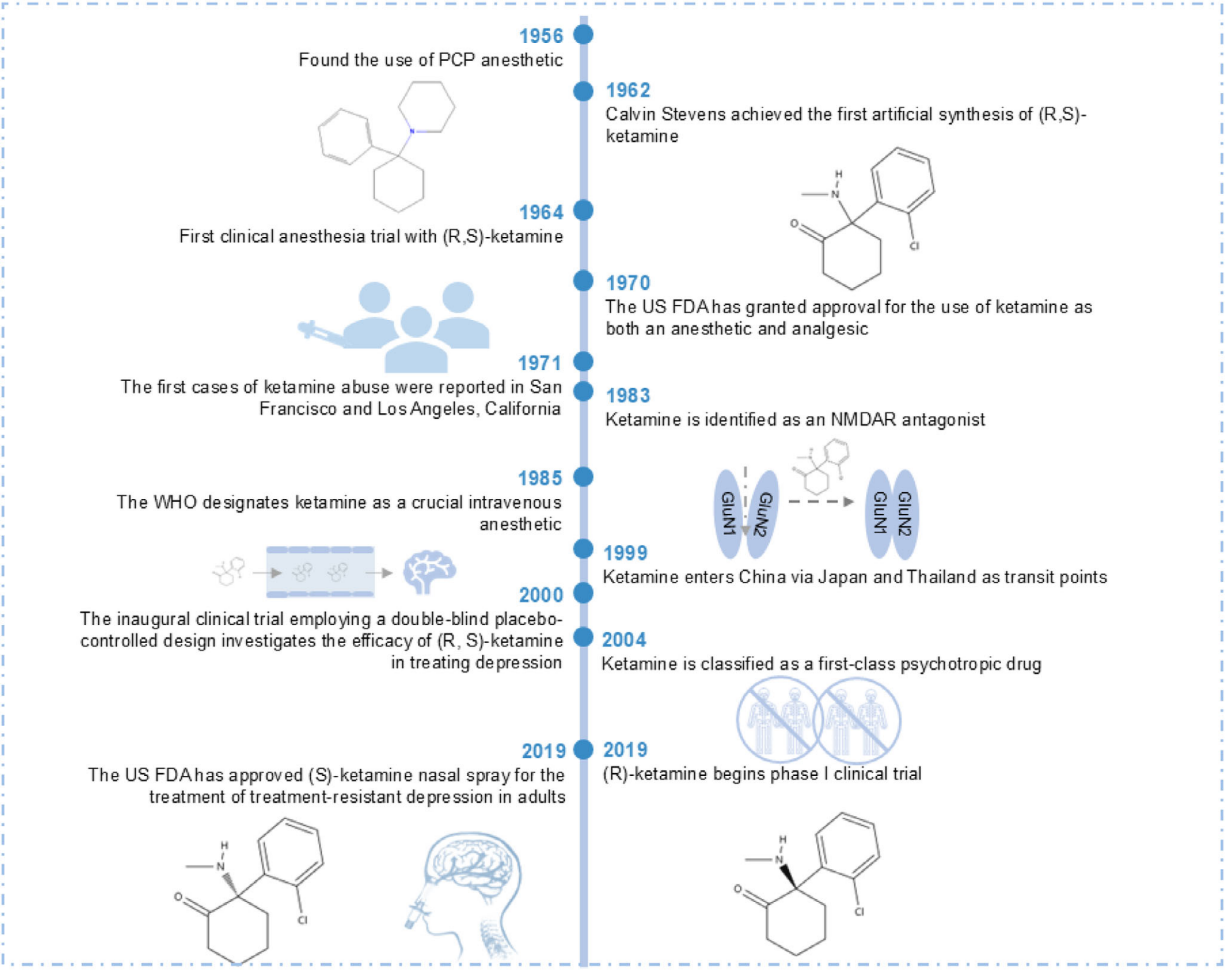
A comprehensive review of the history of Esketamine/ketamine. The first synthesis of ketamine occurred over 50 years ago. In 1956, a substance called phenylcyclohexylpiperidine (PCP) was first synthesized, which is a type of hallucinogenic anesthetic with many side effects. In 1962, American pharmacist Calvin Stevens first synthesized ketamine, which is a safer short‐acting anesthetic compared to PCP. In 1964, ketamine was first used in human trials, demonstrating the effect of "separative anesthesia". Subsequently, in 1970, the US FDA approved ketamine for general anesthesia, and it was widely used in battlefield anesthesia and emergency treatment in the following decade. In 1983, ketamine was discovered to be an NMDAR antagonist. Encouragingly, in 2000, the team led by John Krystal at Yale University in the United States first reported that sub‐anesthetic doses of ketamine could be used to relieve depression. In 2019, the FDA approved esketamine nasal spray for the treatment of treatment‐resistant depression, making it the first new‐type antidepressant in ≈50 years. In conclusion, the transformation of ketamine from a battlefield anesthetic to a versatile agent for mental illnesses and chronic diseases indicates that it has certain development potential.

S‐ketamine and R‐ketamine are two enantiomers of ketamine. S‐ketamine is also known as esketamine. Esketamine has a pain‐relieving and sedative effect that is four times stronger than levorotatory ketamine. Esketamine has a high clearance rate and a rapid elimination rate.^[^ ^14^, ^15^, ^16^ ^]^ At low doses, it can achieve the same anesthetic and analgesic effects as levorotatory ketamine, with higher bioavailability and better safety.^[^ ^17^, ^18^ ^]^ It is more suitable for certain special patients in clinical applications.^[^ ^19^, ^20^, ^21^, ^22^ ^]^ However, compared with R‐ketamine, the dissociation effect, hemodynamic effect, and the possibility of abuse of esketamine still need to be carefully considered^[^ ^23^ ^]^ (**Figure** **1**).

This review will discuss the application of esketamine and ketamine from four aspects: classical application,^[^ ^24^, ^25^, ^26^ ^]^ antidepressant effect,^[^ ^27^, ^28^, ^29^ ^]^ anti‐tumor effect,^[^ ^30^, ^31^, ^32^, ^33^, ^34^ ^]^ and immune‐inflammatory effect.^[^ ^35^, ^36^, ^37^, ^38^, ^39^, ^40^, ^41^ ^]^ We will separately discuss the mechanism of action, specific clinical applications, and current potential limitations of esketamine and ketamine in these four aspects.

## 2 Mechanism and Clinical Application of Esketamine/Ketamine in Analgesia, Sedation, and Anesthesia

The classic applications (analgesia, sedation, and anesthesia) of esketamine and ketamine are mainly based on their unique “separate anesthesia” property. “Separate anesthesia”^[^ ^42^ ^]^ means that although the patient appears to be conscious and can maintain some reflexes, their consciousness is separated from their physical sensations and the external environment, and thus they cannot feel pain.^[^ ^43^ ^]^ It is precisely this mechanism that gives it its uniqueness in providing analgesia, sedation, and combined anesthesia.

### 2.1 Mechanism of Esketamine/Ketamine in Analgesia, Sedation, and Anesthesia

#### 2.1.1 Mechanism of Esketamine/Ketamine in Analgesia

**Figure 2:**
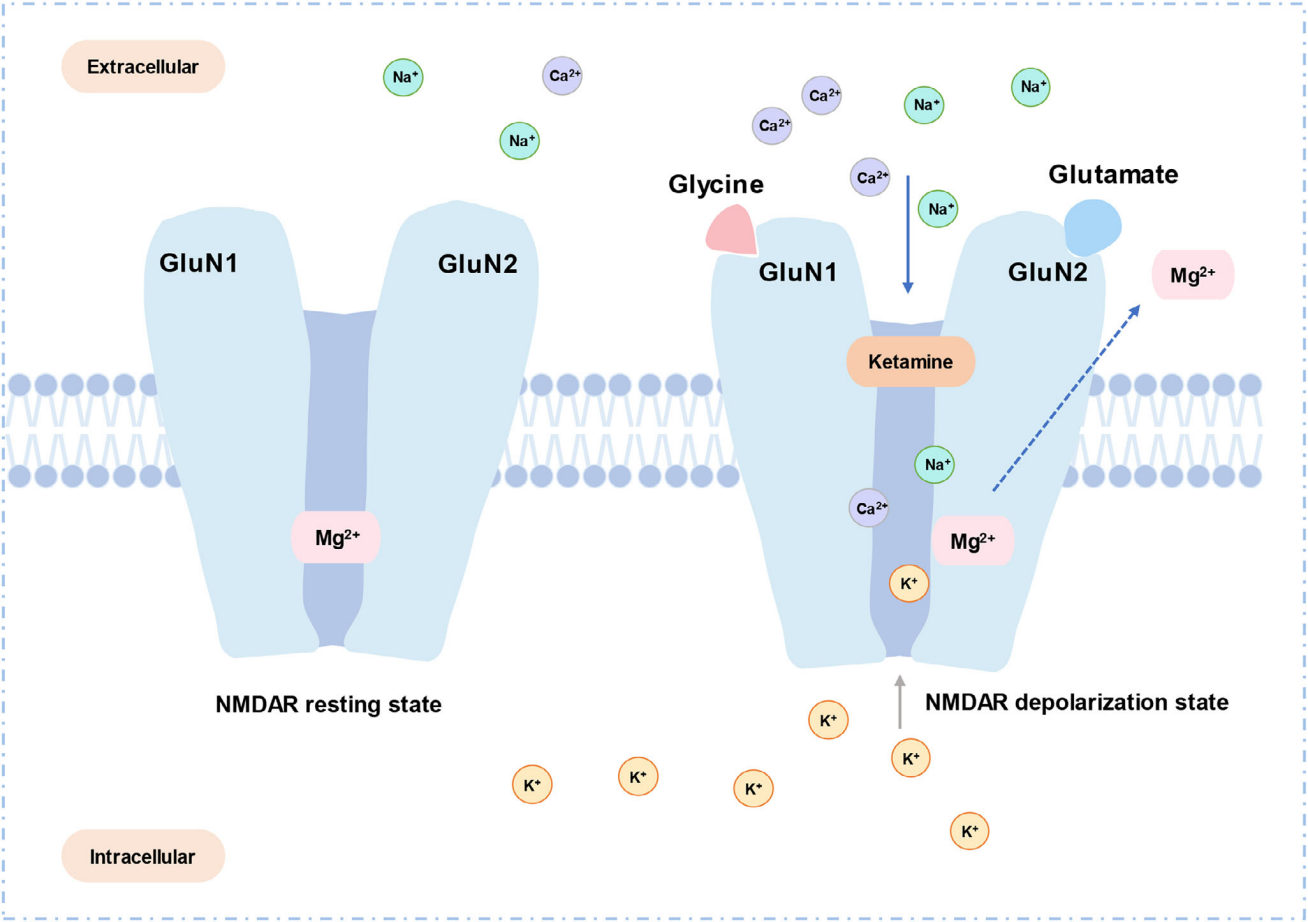
Molecular architecture of the NMDA receptor. NMDAR is a transmembrane ion channel protein composed of four subunits (typically 2 GluN1 and 2 GluN2), which together form a channel selective for cations. This receptor has two important ligand‐binding sites: the glycine‐binding site on the GluN1 subunit and the glutamate‐binding site on the GluN2 subunit. In the resting state, even if both sites are simultaneously occupied by their respective agonist ligands, the channel remains blocked by Mg^2^⁺ ions and remains closed. When NMDAR postsynaptic depolarization releases Mg^2^⁺ ions, glutamate and glycine co‐agonists bind to the receptor, opening the NMDAR channel, leading to increased Ca^2^⁺ influx, and subsequently activating downstream second messengers. Ketamine and its derivatives can inactivate the receptor and inhibit downstream signaling pathways by binding to the PCP site within the channel.

The analgesic mechanism of esketamine and ketamine involves multiple receptors and channels. Esketamine and ketamine exert analgesic and anti‐hyperalgesic effects by inhibiting NMDAR. NMDAR is an important subtype of ionotropic glutamate receptors, composed of two GluN1 and two GluN2 subunits. Dysregulation of NMDAR function is closely associated with the pathogenesis of various diseases, including neurodegenerative diseases, chronic pain, mental disorders, and ischaemic brain injury.^[^ ^44^, ^45^ ^]^ When neurons are in a resting state, NMDAR channels are blocked by Mg^2+^. When glutamate and glycine (co‐excitatory agents) bind to NMDAR simultaneously, it triggers a conformational change, activating NMDAR and releasing Mg^2+^, which leads to increased Ca^2+^ influx and activates downstream second messengers, producing substances such as Nitric oxide (NO) and prostaglandins.^[^ ^44^, ^45^, ^46^, ^47^ ^]^ Excessive production of NO directly inhibits gamma‐aminobutyric acid (GABA) neurotransmission, thereby reducing presynaptic inhibition. NO can also promote the release of presynaptic glutamate, enhancing harmful signal transmission.^[^ ^48^, ^49^, ^50^, ^51^, ^52^ ^]^ Prostaglandins can also amplify the transmission efficiency of pain signals.^[^ ^50^, ^53^ ^]^ In summary, esketamine and ketamine disrupt the function of NMDAR and inhibit downstream signaling pathways to exert their analgesic effects (**Figure** **2**).

**Figure 3:**
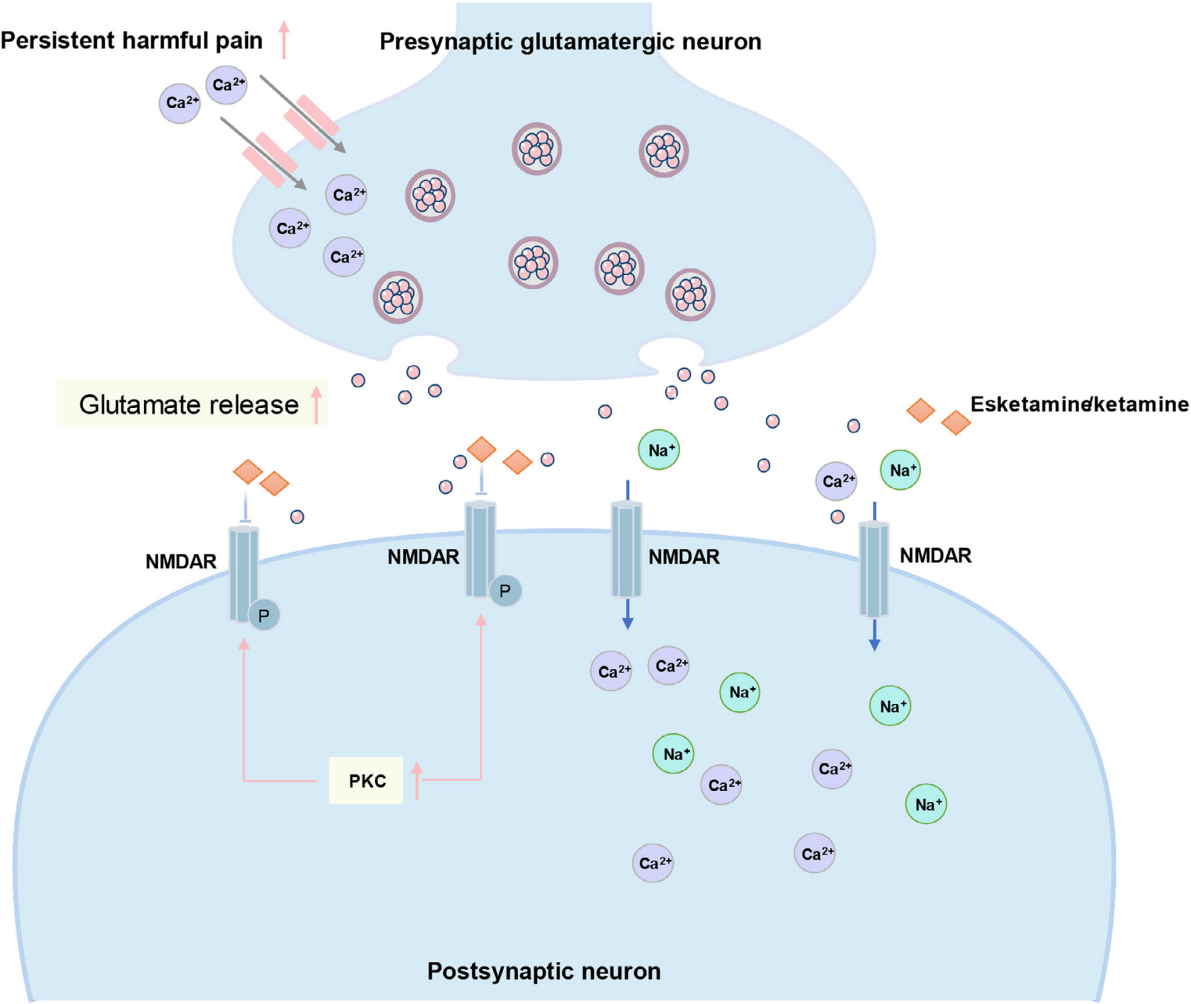
Mechanisms of NMDAR in central sensitization. Under normal physiological conditions, NMDAR serves as a key mediator in glutamatergic neurotransmission, participating in the signaling of noxious stimuli. However, when subjected to severe, persistent pain, there is a significant increase in calcium ion influx into presynaptic neurons, leading to enhanced glutamate release. NMDAR on postsynaptic neurons becomes overly activated, triggering calcium and sodium ion influx. Intracellular calcium overload activates protein kinase C (PKC), promoting phosphorylation of NMDARs. Phosphorylation reduces the inhibitory effect of magnesium ions on NMDARs, significantly prolonging their open time. However, ketamine and esketamine can block NMDARs and the aforementioned pathological changes.

Besides the well‐recognized direct antagonistic effect on NMDAR, esketamine and ketamine can also suppress the sensitization process of the central nervous system. Sensitisation of the central nervous system manifests as abnormal increases in the excitability of spinal dorsal horn neurons, leading to two symptoms: abnormal pain and hyperalgesia.^[^ ^46^ ^]^ It is worth noting that abnormal activation of NMDAR is associated with spinal neuron hyperexcitability and the development of chronic pain.^[^ ^46^ ^]^ In a state of pain, calcium ion concentration within neurons increases, thereby activating protein kinase C (PKC). PKC phosphorylates NMDAR, reducing Mg^2+^ blockade at the resting membrane potential and making the channel more likely to open.^[^ ^54^, ^55^ ^]^ PKC can also influence receptor localisation and function at synapses by regulating postsynaptic dense region cytoskeletal proteins of NMDAR, particularly postsynaptic density protein‐95 (PSD‐95),^[^ ^46^ ^]^ thereby participating in the processing of spinal cord injury information.^[^ ^55^, ^56^, ^57^ ^]^ Esketamine and ketamine can also block NMDAR and the aforementioned pathological changes, thereby reducing the amplification of sensitisation reactions in the central nervous system (**Figure** **3**).

The opioid receptor system may also mediate the analgesic effects induced by esketamine and ketamine. It is established that the analgesic effect of ketamine is significantly attenuated in µ‐opioid receptor knockout mice.^[^ ^58^ ^]^ Researchers have found that naloxone is a selective µ and δ opioid receptor antagonist that can reverse the peripheral analgesic effects induced by ketamine in a dose‐dependent manner.^[^ ^59^ ^]^ These findings suggest that opioid receptors may be involved in the analgesic effects of ketamine. Ketamine may exert its analgesic effects by preventing opioid‐induced hyperalgesia (OIH).^[^ ^60^, ^61^, ^62^ ^]^ The NMDAR antagonist ketamine can block and reverse the hyperalgesia that occurs after acute injection and continuous infusion of opioid drugs.^[^ ^61^, ^63^ ^]^ This mechanism explains that the combined use of ketamine and opioid drugs results in a synergistic analgesic effect and reduces the dosage of opioid drugs.^[^ ^11^, ^64^, ^65^, ^66^, ^67^ ^]^

The metabolites of ketamine, desmethylketamine (80%), hydroxydesmethylketamine (15%), and hydroxyketamine (5%), appear to contribute to the ultimate analgesic effect of ketamine.^[^ ^68^, ^69^, ^70^, ^71^, ^72^, ^73^, ^74^, ^75^ ^]^ Esketamine may treat neuropathic pain by inhibiting large‐conductance Ca^2+^‐activated potassium channels (BK channels). Excessive activation of spinal microglia following peripheral nerve injury is associated with the development of neuropathic pain. Esketamine inhibits the excessive activation of microglia by blocking BK channels, thereby alleviating neuropathic pain.^[^ ^76^, ^77^ ^]^

#### 2.1.2 Mechanism of Esketamine/Ketamine in Sedation

The sedative and hypnotic effects of esketamine and ketamine may be attributed to its rapid blockade of hyperpolarized activated cyclic nucleotide‐gated cation (HCN‐1 receptor) channels. HCN channels (particularly the HCN‐1 subtype) are vital for regulating neuronal activity in brain regions such as the thalamus, hippocampus, and cortex. When blocked by ketamine‐type drugs, HCN channels reduce the I_h_ current (a pacing current generated by the activation of cell membranes during hyperpolarisation), which reduces neuronal excitability and rhythmic firing, leading to sedative and hypnotic effects.^[^ ^78^, ^79^, ^80^, ^81^ ^]^ It also may promote slow wave sleep patterns by reducing cholinergic activity in the pontine region.^[^ ^82^ ^]^ In the emergency department, patients with rapid agitation are given a muscle injection of 5 mg kg^−1^ of ketamine, which provides adequate sedation in a significantly shorter time than a combination of intramuscular midazolam and haloperidol.^[^ ^83^ ^]^ The level of adenosine triphosphate (ATP) in the neurons of patients with traumatic brain injury is decreased. This decrease subsequently leads to elevated levels of glutamate in the body. Ketamine has been proven to reduce the level of glutamate in the brain to a certain extent and to inhibit the activation of microglia cells.

#### 2.1.3 Mechanism of Esketamine/Ketamine in Anesthesia

Esketamine and ketamine mainly exert anesthetic effects by inhibiting NMDAR. When the NMDAR channels open and form hydrophobic bonds and hydrogen bonds, esketamine and ketamine interact with specific amino acids (leucine 642 on GluN2A, leucine 643 on GluN2B, and asparagine 616 on GluN1) in the transmembrane domain of the ion channel,^[^ ^87^ ^]^ thereby altering the structure of the ion channel, inhibiting the high permeability of Ca^2+^, reducing the opening time and frequency of the channel, and preventing the activation of neurons required for the state of consciousness, thus achieving the anesthetic effect. In local anesthesia, esketamine can exert inhibitory effects on sensation and movement by blocking Na⁺ channels.^[^ ^88^, ^89^, ^90^ ^]^

The antagonistic effect of esketamine on NMDAR may also activate α‐amino‐3‐hydroxy‐5‐methyl‐4‐isoxazolepropionic acid receptor (AMPAR) at the same time. Studies have shown that differences in amino acids at the key sites of AMPAR may lead to the disruption of the spatial structure of ketamine, and the spatially sensitive hydrophobic interactions also weaken accordingly. This may enable esketamine to selectively bind to NMDAR rather than AMPAR.^[^ ^87^ ^]^ The study found that blocking NMDAR would reduce the release of GABA by γ‐aminobutyric acid (GABA)‐ergic interneurons, leading to disinhibition of glutamatergic neurons and thereby indirectly activating AMPAR.^[^ ^91^ ^]^ The metabolite of esketamine (2R,6R)‐hydroxydexamethorphan can also continuously activate AMPAR.^[^ ^92^ ^]^ The enhanced signal after AMPAR activation can lead to an increase in synaptic transmission in the thalamus and cortex. This might partly explain the different characteristics of the anesthesia state induced by ketamine compared to those induced by other anesthetics.

### 2.2 Clinical Application in Analgesia, Sedation, and Anesthesia

Esketamine and ketamine are among the few anesthetics that possess both analgesic and sedative effects. They can provide stable hemodynamics, with relatively mild respiratory depression and significantly reduced adrenal suppression. Their proven anesthetic, sedative, and analgesic efficacy supports diverse roles—from a single‐agent for short surgeries to a key adjunct in regional and combined anesthesia, particularly in vulnerable populations like pediatric, obstetric, and elderly patients.

#### 2.2.1 Clinical Application in Analgesia

Several current studies have shown that administering esketamine during surgery can significantly reduce the consumption of postoperative opioid drugs (e.g., fentanyl and oxycodone),^[^ ^99^, ^103^ ^]^ effectively alleviating acute pain after surgeries involving the abdomen, weight loss, and breast cancer.^[^ ^100^, ^104^ ^]^ It can also lower the incidence of propofol injection pain^[^ ^102^ ^]^ and potentially offer benefits in preventing chronic pain after breast cancer surgery.^[^ ^107^ ^]^ Furthermore, in the emergency management of acute pain, the analgesic effect of intranasal administration of ketamine is not inferior to that of morphine.^[^ ^110^, ^111^ ^]^ However, there is also inconsistency in the evidence, with some studies indicating that the analgesic effect of low‐dose ketamine on acute pain is not significant, or suggesting that its analgesic effect is only temporary.^[^ ^101^, ^105^ ^]^ Overall, under appropriate dosing regimens, this class of drugs can provide safe and effective postoperative pain relief while reducing the side effects associated with opioids. However, their efficacy may be influenced by various factors such as dosage, type of surgery, and timing of administration.

#### 2.2.2 Clinical Application in Sedation

Esketamine and ketamine have demonstrated various positive effects in perioperative sedation and hypnosis. Studies have shown that esketamine can improve patients' postoperative sleep quality, alleviate short‐term anxiety and depression, reduce postoperative pain and stress responses, and effectively shorten the postoperative bed rest time.^[^ ^114^, ^115^, ^116^ ^]^ In terms of controlling sedation and agitation, the duration of adequate sedation after intramuscular injection of ketamine may be relatively short,^[^ ^84^ ^]^ but it significantly outperforms haloperidol/lorazepam in controlling acute agitation, and performs well in the overall sedation experience.^[^ ^117^ ^]^ From a safety standpoint, it is not only associated with a decrease in the incidence of postoperative nausea and vomiting. Moreover, the majority of patients reported either no pain or only mild pain, without any significant related complications. Although it may, to a certain extent, prolong the recovery time of consciousness and orientation.^[^ ^118^, ^119^ ^]^

#### 2.2.3 Clinical Application in Anesthesia

Esketamine and ketamine for perioperative anesthesia can be applied in various populations and surgical scenarios. In the pediatric field, it has been proven to be safe and effective for anesthesia induction during coronary heart disease surgeries in children, and can provide satisfactory emergency sedation for children with forearm fractures.^[^ ^93^, ^94^ ^]^ In obstetric cesarean section procedures, the use of small doses can stabilize the vital signs of the mother, alleviate pain, and pose no threat to the safety of the newborn.^[^ ^95^ ^]^ Its pharmacological advantage lies in stimulating the sympathetic nerve, effectively counteracting the inhibition of heart rate and the drop in blood pressure caused by propofol and opioid drugs, thereby maintaining a more stable hemodynamic state. This feature has also been verified in elderly patients. The combined use of propofol not only stabilizes blood pressure during the operation, accelerates cognitive recovery, but also reduces inflammatory responses and the risk of side effects, and does not affect early rehabilitation after knee joint replacement.^[^ ^96^, ^97^ ^]^

A recent study of patients undergoing painless gastroscopy in China made a comparison of the pharmacokinetic profile and safety of esketamine and ketamine.^[^ ^98^ ^]^ The results demonstrated that a single administration of 0.5 mg kg^−1^ of esketamine or 1 mg kg^−1^ of ketamine was well tolerated by patients, with no occurrence of serious adverse reactions. Esketamine has been shown to have a shorter recovery time and directed recovery time, indicating its potential clinical advantages. In other painless procedures, such as endoscopic retrograde cholangiopancreatography (ERCP), hysteroscopy, laparoscopy, and fiberoptic bronchoscopy, esketamine can not only relieve patients' fear and anxiety, but also reduce the intensity of pain and the need for opioids in the short term after surgery. This table summarizes the clinical trial results of ketamine and esketamine in the applications of anesthesia, sedation‐hypnotics, and analgesia in recent years, aiming to provide a reference for clinical practice (**Table** **1**).

**Table 1:** Clinical Applications of Esketamine and Ketamine.

Experiment object	Sample size	Drug dosage	Follow‐up duration	Result	Bias risk scores	Reference
Clinical Application of Pain Management						
Thyroidectomy patients	Con:Exp/30:30	IV ESK 0.5 mg kg^−1^ prior to incision + maintenance dose ESK 0.24 mg kg^−1^h^−1^	Within 24 h after the operation	Intraoperative administration of esketamine reduces perioperative fentanyl consumption and postoperative pain.	Low risk	[99]
Weight loss surgery patients	Con:Exp/34:34	IV ESK 0.2 mg kg^−1^ + maintenance dose ESK 0.2 mg kg^−1^h^−1^	Within 12 h after administration	The use of esketamine during surgery can alleviate acute postoperative pain in patients undergoing weight loss surgery.	Low risk	[100]
Vestibular schwannoma resection surgery	Con:Exp/45:45	IV ESK 0.2 mg kg^−1^	Within 24 h after the operation	Low‐dose ketamine administered during surgery did not significantly alleviate acute pain in patients	Low risk	[101]
Patients with pain from propofol injection	Con:Exp1: Exp2/33:33:35	30 s before injecting propofol 0.15 mg kg^−1^ of ESK	Within 24 h after the injection	It can reduce the incidence of pain associated with propofol injections and provide more stable circulation for patients after anaesthesia induction.	Low risk	[102]
Patients undergoing lumbar fusion surgery	Con:Exp1:Exp2: Exp3/25:25:25:25	Three groups of ESK: 0.25 mg/mL/0.5 mg/mL/0.75 mg/mL+ hydrocodone 1 mg/mL	Within 24 h after the operation	Within 24 h postoperatively, the total consumption of oxycodone in the experimental group was significantly reduced, with no additional adverse reactions.	Low risk	[103]
Patients undergoing lumbar fusion surgery	Con:Exp1: Exp2/66:66:66	Group 1:Preoperative IV ESK 0.5 mg kg^−1^+ maintenance dose ESK 0.12 mg kg^−1^h^−1^. Group 2: Preoperative IV ESK 0.5 mg kg^−1^ + maintenance dose ESK 0.6 mg kg^−1^h^−1^.	Within 48 h after the operation	Four hours after surgery, the pain scores in the experimental group were lower than those in the control group, with statistical significance, and pain relief was temporary.	Some concerns	[104]
Arthroscopic anterior cruciate ligament repair	Con:Exp/25:25	IV injection of 0.5 mg kg^−1^ ESK + 2 µg kg^−1^h^−1^ 5 min before incision	1, 24, 48, 72, and 120 h after the operation	It has no effect on postoperative pain relief	Low risk	[105]
Thoracoscopic radical resection of lung tumor	Con:Exp/79:80	Epidural 0.25 mg kg^−1^ ESK +10 mL0.2% ropivacaine before incision, IV injection 0.125 mg kg^−1^ ESK when needed, postoperative controlled epidural analgesia 25 mg ESK +0.15% ropivacaine	The incidence of chronic pain 3 months after the operation	It can reduce the incidence of postoperative mild chronic pain and side effects	Some concerns	[106]
Chronic pain after breast cancer surgery	Con:Exp/45:45	Oral 150 mg pregabalin + IV controlled analgesia pump: 25 mg kg^−1^ ESK +100 µg sufentanil +4 mg tropisetron	The incidence of chronic pain at 3 months and 6 months after the operation	It can effectively prevent chronic pain after breast cancer surgery, improve acute postoperative pain, and reduce postoperative opioid consumption	Low risk	[107]
Analgesia after cesarean section	Con:Exp/166:161	Self‐controlled epidural analgesic pump: 0.5 mg kg^−1^ ESK + Sufentanil 2 µg kg^−1^+ tropisetron 10mg	On the 3rd day, the 14th day, and the 28th day after the operation	IV injection of esketamine 0.01 mg kg^−1^h^−1^ at 14 days after surgery could reduce the incidence of postpartum abdominal pain, and the pain was significantly relieved within 48 days after delivery, and the incidence of adverse reactions did not increase	Some concerns	[108]
Postoperative analgesia in children with hypospadias	Con:Exp/77:77	IV injection of 0.3 mg kg^−1^ ESK +0.15 mg kg^−1^h^−1^ maintained ESK + sacral epidural block	Postoperative 0, 2, 6, 12, 24, 36 and 48 h	It provides safe and effective postoperative analgesia	Some concerns	[109]
Outpatient traumatic pain patients	Con:Exp/123:128	IV ketamine 20 mg, followed by 10 mg every 5 min	From before administration to 30 min after administration	Compared with morphine, intravenous ketamine demonstrated non‐inferiority in pain relief.	Some concerns	[110]
MMPR patients	Con:Exp/156:144	Infusion of sub‐dissociative doses of ketamine	From 24 to 72 h after admission	Adding sub‐dissociative ketamine infusion to oral MMPR reduces opioid exposure in critically ill patients.	Some concerns	[111]
Emergency department acute pain patients	Con:Exp/75:75	IV 0.3 mg kg^−1^ ketamine or 0.75 mg kg^−1^ IN ketamine	At 15, 30, 60, 90, and 120 min after administration	Both treatments reduced clinically significant pain scores at 30 min with no significant difference between them.	Low risk	[112]
Patients with neuropathic pain	Con:Exp/50:50	Receive IN ketamine (1 mL = 50 mg) 5 min before surgery.	45 min after the operation	IN Ketamine can effectively relieve pain in patients with acute pain without increasing significant side effects.	Low risk	[113]
Clinical Applications of Sedation and Hypnosis						
Gynecological laparoscopic surgery	Con:Exp/91:92 Con:Exp/38:39	IV injection of 0.3 mg kg^−1^h^−1^ ESK + dexmedetomidine 0.1–0.3 µg kg^−1^min^−1^+ propofol 5–7 mg kg^−1^h^−1^	On the first and third days after the operation/ Within 48 h after the operation	Improved postoperative sleep quality; The incidence of postoperative nausea and vomiting was lower, and the recovery time of awakening and orientation was prolonged	Low risk Low risk	[114, 115]
Elderly hip replacement	Con:Exp/75:75	IV injection of 2.5 mg kg^−1^ ESK	Three days after the operation, one week after the operation, one month after the operation	Reduce short‐term anxiety and depression, relieve postoperative pain and stress response, shorten postoperative bed time	Low risk	[116]
Agitated patient in the emergency department	Con:Exp/40:39	5 mg kg^−1^ ketamine IM injection	Within 30 min after administration	The duration of adequate sedation following IM injection of ketamine was significantly shorter than that in the control group	Low risk	[84]
Acute agitated patients in the emergency department	Con:Exp/45:41	4 mg kg^−1^ IM injection or 1 mg kg^−1^ IV injection of ketamine	Within 5 min and 15 min after administration	Ketamine is significantly superior to haloperidol/lorazepam in the initial control of acute agitation.	High risk	[117]
Patients with AF undergoing PFA	Con:Exp/33:33	Administer ketamine adjuvant (1 mg kg^−1^) ≈5 min prior to PFA administration.	Within 1 h after administration	All patients reported no pain or mild pain. No major surgical or anesthesia‐related complications were reported.	Not applicable	[118]
Endoscopy patient	Con:Exp/33:33	1 mg dose of midazolam and ketamine	The duration of the operation	Ketamine performed well in overall sedation experience and all analytical categories.	Some concerns	[119]
Clinical application of Anesthesia						
Unilateral total knee replacement	Con:Exp/40:40	IV injection 0.2 mg kg^−1^ ESK	Before anesthesia induction, during tracheal intubation, and at 1 and 5 min; during skin incision and at 1 and 5 min	The hemodynamics induced by low dose anesthesia was more stable and had no adverse effect on the quality of early postoperative recovery	High risk	[97]
Hysteroscopy and coning of cervix	Con1:Con2: Exp/49:49:45	IV injection of 0.5 mg kg^−1^h^−1^ ESK+ afentanil 10–20 µg kg^−1^	Within 24 h after the operation	The use of opioids was reduced, and the stay time in the anesthesia recovery room was increased in the low‐dose opioid group	Low risk	[120]
Lumbar fusion surgery	Con:Exp1:Exp2: Exp3/25:25:25:25	IV injection of 0.75 mg mL^−1^ ESK +1 mg mL^−1^ oxycodone	Within 24 h after the operation	Postoperative consumption of oxycodone was significantly lower and there were no serious adverse reactions	Low risk	[103]
Strabismus surgery for children	Con:Exp1: Exp2/59:61:60	IV infusion of 0.25 mg kg^−1^ ESK +0.1 µg kg^−1^ sufentanil +3 mg kg^−1^ propofol	Before induction, after induction for 1 min but before the laryngeal mask is inserted, immediately after the laryngeal mask is inserted, 2 min after the laryngeal mask is inserted	The maximum dose of 0.5 mg kg^−1^ combined with propofol and sufentanil did not increase intraocular pressure from baseline	Low risk	[121]
Open reduction and internal fixation of lower limbs in children	Con:Exp/60:60	IV injection 0.5 mg kg^−1^ ESK + sciatic nerve and femoral nerve block	Before anesthesia, and 10, 20, and 30 min after anesthesia	It is better than general anesthesia combined with ultrasound‐guided nerve block anesthesia to reduce the incidence of complications	High risk	[122]
Fiberbronchoscopy	Con:Exp/40:40	IV injection 0.15 mg kg^−1^ ESK + propofol 1 mg kg^−1^	During the operation and within the first 10 min after the operation	Low dose combined propofol is completely effective in elderly patients, and the respiratory cycle is stable	Low risk	[123]
Endoscopic retrograde cholangiopancreatography	Con:Exp/79:83	IV injection 150 µg kg^−1^ ESK + propofol plasma effective concentration 1.5 µg mL^−1^	During the intraoperative endoscopic retrograde cholangiopancreatography procedure	Compared with afentanil, the total amount of propofol can be reduced without affecting the recovery time and patient physician satisfaction	Low risk	[85, 86]
Painless gastroenteroscopy	Exp1:Exp2/16:16 Con:Exp/51:51	IV injection 0.5 mg kg^−1^ ESK IV injection of 0.3 mg kg^−1^ ESK + propofol	Within 24 h after administration/During the operation and within 24 h after the operation	A single injection is safe and reliable, and patients recover faster; Compared with dezocine, the total amount of propofol can be reduced with fewer adverse reactions	Some concerns Low risk	[98, 124]
Painless abortion	Con:Exp1:Exp2: Exp3/45:45:44:44	IV injection of 0.2‐0.3 mg kg^−1^ ESK + propofol 2 mg kg^−1^	During the operation and within 24 h after the operation	A single dose of 0.25 mg kg^−1^ can effectively reduce the incidence of hypotension and adverse reactions, and reduce the dosage of propofol	Low risk	[125]

IV, Intravenous; ESK, Esketamine; EGG, Electroencephalogram; MMPR, Oral multimodal pain management; IN, Intranasal; IM, Intramuscular; AF, Atrial Fibrillation; PFA: Pulsed Field Ablation;Con, Control group; Exp, Experimental group.

### 2.3 Potential Limitations

The clinical use of esketamine and ketamine is associated with some side effects; however, most are transient and typically resolve within a few hours of administration. In clinical anesthesia, these two drugs can cause an increase in heart rate and blood pressure by activating the sympathetic nervous system and antagonising cholinergic M receptors.^[^ ^126^, ^127^ ^]^ Intravenous infusion of esketamine (1 mg kg^−1^) can increase the mean arterial pressure of healthy volunteers by 10 mmHg and heart rate by 10 bpm, with these haemodynamic changes being most pronounced within 30 min after administration. It should be avoided in patients with acute brain injury.^[^ ^128^ ^]^ They may cause an increase in pulmonary vascular resistance and an increase in respiratory secretions, so there is a risk of respiratory depression when administered rapidly. Some patients who recover after anesthesia may have varying degrees of disorientation, euphoria, or obvious delirium, with an incidence rate ranging from 5% to 30%. As well as temporary double vision, increased intraocular pressure, as well as mild nausea and vomiting and other adverse reactions.

On the other hand, the widespread promotion of esketamine and ketamine also faces challenges. From the regulatory perspective, the strict control of them as a class of psychotropic drugs (e.g., the need for full supervision by certified institutions during use) ensures safety, but significantly limits clinical accessibility and increases medical costs. The high cost of treatment and the lack of extensive medical insurance coverage have also imposed a heavy financial burden on patients. From a safety perspective, the inherent risks of abuse and addiction require the establishment of strict risk management systems. Therefore, how to balance their significant therapeutic effects with these potential risks is a core problem that needs to be jointly solved in the future.

## 3 Mechanism and Clinical Application of Esketamine/Ketamine in Anti‐Depressant

Currently, depression has become a common mental illness, especially treatment‐resistant depression. But traditional antidepressants such as tricyclic, tetracyclic, and monoamine oxidase inhibitors often take a long time to work, and long‐term use can lead to dependence and withdrawal (e.g., dizziness, nausea, anxiety, and insomnia).^[^ ^129^ ^]^ A study conducted in 2000 revealed that sub‐anesthetic doses of ketamine could induce rapid and significant antidepressant effects, marking another major breakthrough following traditional monoamine therapy.^[^ ^4^ ^]^

Therefore, we reviewed the mechanisms of action of esketamine and ketamine as rapid‐acting antidepressants, which involve glutamate‐related receptors, including inhibition of GABAergic interneurons via NMDAR (the disinhibition hypothesis), synaptic NMDAR inhibition, inhibition of spontaneous NMDAR‐mediated neurotransmission, inhibition of NMDAR‐dependent lateral raphe neuron burst firing, and the effects of ketamine metabolites; as well as receptors beyond glutamate, including the monoamine system, the nitric oxide (NO) system, and the opioid receptor system.^[^ ^92^, ^130^, ^131^, ^132^, ^133^, ^134^ ^]^ We have also summarized the current efficacy of ketamine and esketamine in treating depression and related suicidal ideation.^[^ ^135^, ^136^, ^137^, ^138^, ^139^ ^]^ As well as the potential limitations (including individual tolerance and potential side effects).

### 3.1 Mechanism of Esketamine/Ketamine in Anti‐Depressant

#### 3.1.1 Antidepressant Mechanism of Esketamine/Ketamine Mediated by Glutamate‐Related Receptors

At present, the comprehension of the rapid and sustained antidepressant effects of esketamine and ketamine is predominantly derived from investigations focusing on glutamate‐related receptors. NMDAR is a glutamate ion channel receptor that is regulated by both voltage and receptor ligands. It plays a key role in the transmission of excitatory nerve impulses in the central nervous system.^[^ ^87^, ^140^ ^]^ Among them, the disinhibition hypothesis is one of the main hypotheses explaining the antidepressant effects of ketamine. It involves the inhibition of gamma‐aminobutyric acid (GABA) interneurons. A crucial subtype of these GABAergic interneurons, known as Somatostatin (SST) interneurons, exerts direct inhibitory and indirect excitatory influences on pyramidal neurons. Previous studies have shown that ketamine can preferentially inhibit the expression of NMDAR on GABAergic interneurons, thereby weakening the activity of inhibitory neurons, enhancing input to excitatory neurons, and ultimately leading to increased glutamate release in pyramidal cells and the medial prefrontal cortex.^[^ ^141^, ^142^, ^143^ ^]^ Increased glutamate release further leads to binding and activation of the postsynaptic AMPAR, thereby enhancing the release of brain‐derived neurotrophic factor (BDNF) and activating the tropomyosin receptor kinase B (TrkB) receptor. This subsequently activates the mechanism target of the rapamycin complex 1 (mTORC1) mechanism target to promote protein synthesis. Studies have found that ketamine exhibits higher affinity for the GluN2D, which is primarily expressed in inhibitory interneurons in the forebrain, further supporting the above inference.^[^ ^144^, ^145^, ^146^, ^147^ ^]^ In a rat animal model, administration of a sub‐anesthetic dose of ketamine resulted in a significant increase in extracellular glutamate levels, accompanied by enhanced glutamate cycling in the prefrontal cortex.^[^ ^141^, ^148^ ^]^ During the use of benzodiazepines (as positive allosteric modulators of GABA receptors), it was observed that the antidepressant effects of ketamine were weakened or delayed, and such combination therapy strategies exacerbated the risk of depression recurrence in Treatment‐Resistant Depression (TRD) patients after treatment.^[^ ^149^, ^150^, ^151^ ^]^

Compared with the disinhibition hypothesis, esketamine and ketamine may also exert its effects by inhibiting extrasynaptic NMDAR. Previous studies have confirmed the presence of NMDARs in extrasynaptic regions, where these receptors are not localized in the postsynaptic dense region but are primarily composed of heterotetrameric complexes containing the GluN2B subunit.^[^ ^152^, ^153^, ^154^ ^]^ It is speculated that NMDARs containing the extrasynaptic GluN2B subunit can be specifically inhibited by ketamine, thereby preventing the strong activation of these receptors induced by glutamate in the environment, which in turn induces the excitation of pyramidal neurons.^[^ ^155^ ^]^ At the same time, glutamate transporter EAAT2, which is expressed on glial cells, can directly regulate the concentration level of glutamate in the environment.^[^ ^156^, ^157^ ^]^ Under normal physiological conditions, the GluN2B subtype selectively activates NMDAR outside the cortical synapse, acting through the mTOR signaling pathway to inhibit protein biosynthesis, thereby ensuring the stability of synaptic function.^[^ ^155^, ^158^, ^159^, ^160^ ^]^ Ketamine can block synapse‐containing GluN2B, inhibit protein synthesis, and induce antidepressant effects through mTOR‐dependent mechanisms. However, adverse side effects have limited the broader application of ketamine, and GluN2B inhibitors have not yet been approved for clinical use. A recent study reported that the absence of another major NMDAR subunit, GluN2A, in the brains of adult mice induces a strong antidepressant‐like response but has limited effects on behaviors mimicking ketamine's psychoactive effects. Treatment with ketamine or MK‐801 rapidly increased the intrinsic excitability of hippocampal principal neurons via GluN2A. These findings suggest that GluN2A may mediates the rapid antidepressant‐like response triggered by ketamine.^[^ ^161^ ^]^

Esketamine and ketamine may indirectly regulate the spontaneous release of glutamate by blocking NMDAR, thus playing an antidepressant role. Spontaneous transmission refers to the process of transmitting neurotransmitters from presynaptic neurons to postsynaptic neurons through random release of synaptic vesicles in the absence of external stimuli. This spontaneous release of glutamate triggers postsynaptic NMDAR activity, generating minimal excitatory postsynaptic currents (mEPSCs), which subsequently inhibit the synthesis of dendritic cell proteins. Research has found that ketamine's inhibition of NMDAR‐mEPSC occurs at physiological levels of Mg^2+^, and this effect is related to ketamine's rapid antidepressant action.^[^ ^162^ ^]^ It is speculated that spontaneous NMDAR‐mediated neurotransmission enhances synaptic neurotransmission through a protein synthesis‐dependent mechanism involving eukaryotic elongation factor 2 kinase (eEF2K) and BDNF, thereby contributing to the antidepressant effects of ketamine.^[^ ^132^ ^]^ An in vitro electrophysiological study has revealed that the perfusion of ketamine significantly diminishes HPC spontaneous NMDAR‐mEPSC transmission in mice subjected to chronic mild stress (CMS). Thus, it inhibited the postsynaptic NMDAR activity induced by spontaneous glutamate release, which ultimately exerted antidepressant effects.^[^ ^132^ ^]^

Esketamine and ketamine may play an antidepressant role by inhibiting the lateral habenular nucleus (LHb). LHb is an important nucleus in the brain that plays an important role in regulating emotions, motivation, reward and decision making. In depressed states, neurons within LHb exhibit abnormal hyperactivity, which in turn sends strong inhibitory signals to dopaminergic neurons in the midbrain, resulting in the suppression of dopaminergic neuron activity. Ketamine can exert a rapid antidepressant effect by inhibiting the abnormal overactivity of NMDAR within LHb and contacting the function of the midbrain dopamine system.^[^ ^163^, ^164^, ^165^, ^166^, ^167^ ^]^ Increased LHb burst firing was observed in rats with congenital learned helplessness and mice subjected to chronic restraint stress. Ketamine has been shown to rapidly reduce LHb burst firing to control levels while producing antidepressant effects.^[^ ^168^ ^]^ These results suggest that NMDAR in LHb may be a pharmacological target for ketamine, and that neurons that fire bursts of activity may be a potential therapeutic target for depression.^[^ ^169^ ^]^ Although the current findings are encouraging, the role of LHb in modulating the antidepressant effects of ketamine has so far been limited to assessing its acute effects (i.e., within 1 h of drug infusion).

**Figure 4:**
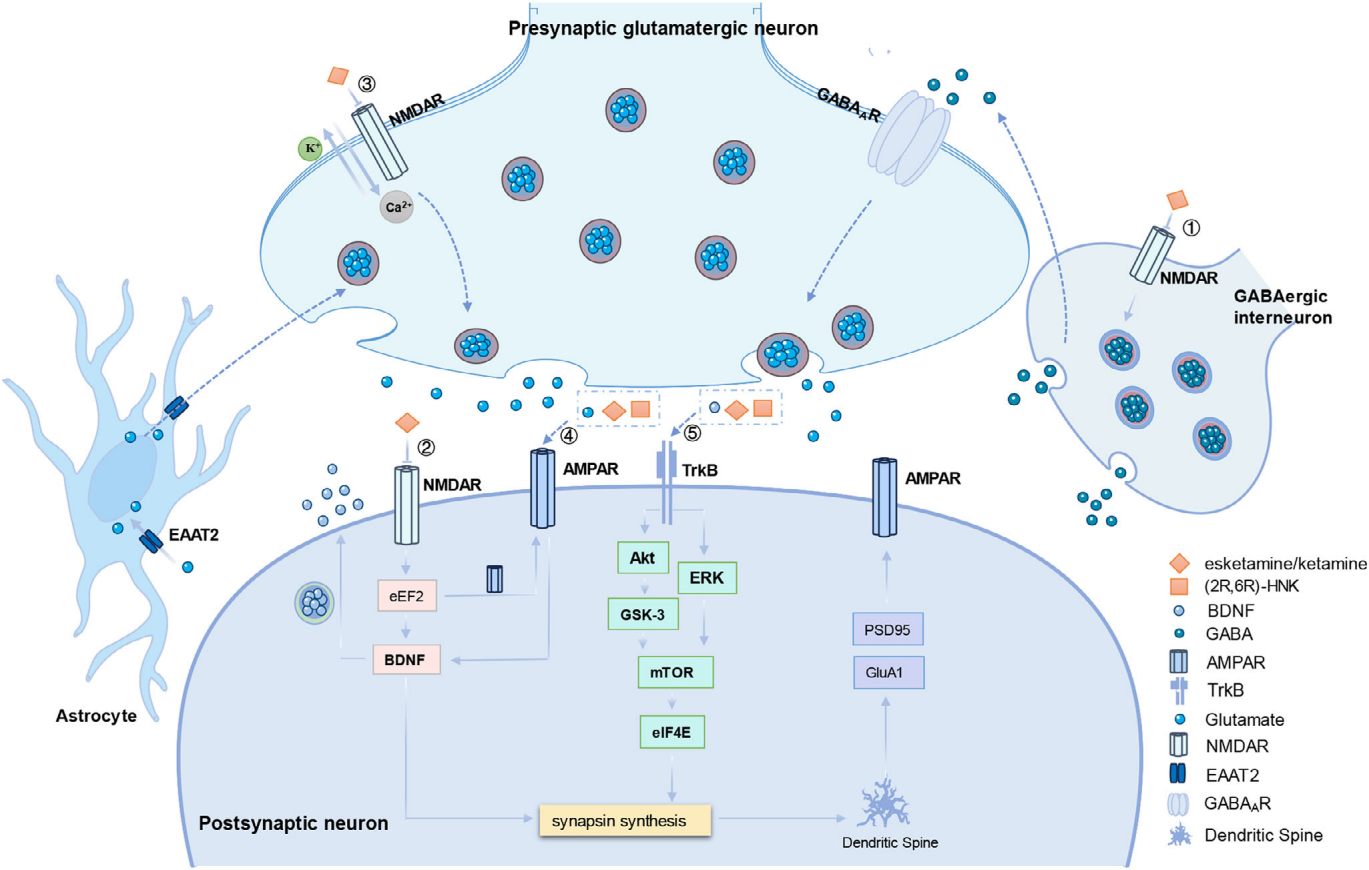
Mechanisms of the glutamatergic system underlying the antidepressant effects of Esketamine/ketamine. ①Disinhibition hypothesis: Esketamin or ketamine blocks NMDAR on GABAergic interneurons, causing GABAergic interneurons to disinhibit and increase discharge frequency, weakens the voltage‐dependent blocking effect of Mg^2+^ on NMDAR, and allows Ca^2+^ to flow into NMDAR, enabling ketamine to enter and selectively bind to NMDAR in GABAergic interneurons. The released glutamate binds to and activates postsynaptic AMPARs, thereby enhancing BDNF release, activating TrkB receptors, and subsequently promoting protein synthesis through the activation of mTORC1. ②Blocking spontaneous NMDAR activation: Esketamin or ketamine inhibits eukaryotic extension factor‐2 (eEF2) kinase (also known as CaMK III) by blocking NMDAR on postsyntic neurons, thereby increasing the translation of BDNF. BDNF protein is released from the postsynaptic neuron and activates the TrkB receptor, which is downstream of TrkB receptor is the signal cascade of extracellular signal‐regulated kinases (ERKs) and protein kinase B (Akt/PKB). The inhibition of GSK‐3 leads to the activation of mammalian target of rapamycin (mTOR), and the activation of mTOR will further activate the phosphorylation of eukaryotic translation initiation factor 4 (eIF4E), increase the number of PSD95 and AMPAR GluA1 subtypes, and promote the formation of frontal cortex synapses and spines.^[^ ^170^, ^171^ ^]^ ③Inhibition of extracellular NMDAR: Ketamine has been proposed to selectively block extracellular GluN2B‐containing NMDARs, which are activated by low levels of ambient glutamate tension regulated by glutamate transporter 1 located on astrocytes. It is hypothesized that inhibiting extracellular GluN2B‐NMDARs can disinhibit mTORC1 function, thereby inducing protein synthesis. ④Esketamin or ketamine and its key metabolites (2S, 6S; 2R, 6R) ‐methylketamine can directly activate AMPAR.^[^ ^131^, ^172^ ^]^ ⑤Esketamin or ketamine and its key metabolites (2S, 6S; 2R, 6R) ‐methylketamine can directly bind to the TrkB, stabilize the dimer structure of TrkB, and increase its expression in synapses, thus enhancing the binding of BDNF to TrkB.^[^ ^173^ ^]^

Although ketamine is a classic NMDAR antagonist, its antidepressant effects may not be entirely dependent on NMDAR. It was found that the active metabolite of ketamine, (2R, 6R)‐hydroxy‐N‐methylketamine [(2R, 6R)‐HNK], has a weaker blocking effect on NMDAR but produces rapid antidepressant effects by increasing the levels of BDNF and GluA1.^[^ ^170^ ^]^ In the mouse experiments, the antidepressant behavior was found to be highly correlated with the higher levels of [(2R,6R)‐HNK] in the brain, not with the levels of ketamine or desmethylketamine.^[^ ^92^ ^]^ This further supports the role of this metabolite in the antidepressant effect of ketamine (**Figure** **4**)

#### 3.1.2 Antidepressant Mechanism of Esketamine/Ketamine Mediated by Non‐Glutamate Related Receptors

The classical monoaminergic hypothesis posits that depression is linked to reduced levels of catecholamines in crucial brain regions, particularly norepinephrine and 5‐hydroxytryptamine (5‐HT).^[^ ^174^ ^]^ A rodent study discovered that subanesthetic doses of ketamine swiftly augmented extracellular 5‐HT levels in the medial prefrontal cortex (mPFC), while local administration of selective 5‐HT1A receptor agonists in the mPFC also induced a rapid and sustained antidepressant effect. Ketamine treatment also increased the activity of dopamine D1‐positive (Drd1) neurons in the medial prefrontal cortex (mPFC). The chemical activation of Drd1 neurons in the mPFC alleviated helpless behavior in mice, and the effects of ketamine were blocked by chemogenetic inhibition of dopamine signaling. These findings suggest that activating Drd1‐positive mPFC neurons via the ventral tegmental area (VTA) is key to restoring stress‐related behavioral responses.^[^ ^175^ ^]^ A study using tryptophan hydroxylase inhibitors found that when ketamine depleted endogenous 5‐HT, its antidepressant effects disappeared.^[^ ^176^ ^]^ Other studies have demonstrated that ketamine can increase 5‐HT levels in the mPFC and activate serotonergic neurons projecting from the mPFC to the dorsal raphe nucleus (DRN) and locus coeruleus (LC), thereby exerting antidepressant effects.^[^ ^177^, ^178^ ^]^ These findings suggest that the mechanism underlying ketamine's antidepressant properties likely involves modulation of the monoaminergic system.^[^ ^179^ ^]^

It has been reported that NO is also associated with the antidepressant effects of ketamine and esketamine. The mechanism involves NMDAR‐dependent activation of neuronal nitric oxide synthase (nNOS), which generates NO. The NO subsequently S‐nitrosylates glyceraldehyde‐3‐phosphate dehydrogenase (GAPDH). The nitrosylated GAPDH then forms a complex with the ubiquitin‐E3 ligase Siah1 and Rheb (a small G protein that activates mTOR), thereby targeting Rheb for degradation by Siah1. In contrast, ketamine has been shown to enhance mTOR signaling by blocking this NO/GAPDH/Rheb signaling cascade.^[^ ^180^ ^]^ However, there is also controversy regarding the role of NO in the antidepressant effects of ketamine. Other researchers have found that administration of the NO donor nitroprusside does not alter the acute antidepressant effects of ketamine in mice.^[^ ^181^ ^]^ It also does not alter the therapeutic response of ketamine in MDD patients 24 h later.^[^ ^182^ ^]^ Therefore, the NO regulatory mechanism still needs to be further clarified.

The research has found that esketamine and ketamine can also exert antidepressant effects through opioid receptors. Studies have shown that pretreatment with the µ‐opioid receptor antagonist naltrexone (which also has mild blocking effects on κ and δ receptors) can prevent the antidepressant effect and suicidal ideation produced by ketamine in patients with TRD.^[^ ^183^, ^184^ ^]^ The research suggests that the µ‐opioid receptor is the target for the antidepressant effect of ketamine,^[^ ^185^ ^]^ and ketamine indeed has a high binding affinity for the µ receptor.^[^ ^186^ ^]^ In animal model experiments, it was found that esketamine has a tendency toward abuse. This is caused by the activation of esketamine and the subsequent downregulation of the brain's µ‐opioid receptors.^[^ ^23^, ^187^ ^]^ Another study has found that ketamine triggers neurophysiological and behavioral responses in the limbic system through the opioid receptor signaling pathway. However, this process is highly gender‐dependent, being significant only in males and driven by sex hormones.^[^ ^188^ ^]^ Clinical studies also support the role of the opioid system in regulating the acute response to ketamine and its subsequent antidepressant effects.^[^ ^189^ ^]^ However, in the depression model rats with impaired innate learning ability, the rescue effect of ketamine on behavior and the activity of the lateral habenula could be blocked by naltrexone, but the µ‐opioid receptor agonist itself had no therapeutic effect. This indicates that the opioid receptor signaling system is involved in the antidepressant effect of ketamine, but it is not the sole or sufficient mechanism.^[^ ^190^ ^]^ Furthermore, there are still some studies that failed to observe that naltrexone reduces the antidepressant effect of ketamine, and they also question the involvement of the opioid receptor system.^[^ ^191^, ^192^ ^]^ This may be related to factors such as the heterogeneity of the patient population (whether there is comorbidity of substance use disorders), the selectivity differences of opioid modulating drugs (the full antagonist naltrexone vs the partial agonist buprenorphine), differences in dosage and administration regimens. In conclusion, there are still different viewpoints regarding the role of opioid receptors in the antidepressant mechanisms of ketamine and esketamine at present.

### 3.2 Clinical Application in Anti‐Depressant

Currently, intranasal and intravenous administration have shown certain efficacy in the TRD and suicidal ideation (SI). Different routes of administration have a significant impact on bioavailability, with the following order from highest to lowest: intravenous injection, intramuscular injection, subcutaneous injection, intranasal administration, oral administration (**Table** **2**). The elimination half‐life of racemic ketamine is ≈2–4 h, while that of esketamine is ≈5 h. The bioavailability of intravenous ketamine can reach 100%, while the bioavailability of intranasal esketamine is ≈30–50%.^[^ ^193^ ^]^ It can circumvent the blood‐brain barrier to mitigate adverse effects in comparison to intravenous administration.^[^ ^194^, ^195^ ^]^ Currently, there is no clear consensus in clinical studies regarding the dose equivalence between intravenous ketamine and intranasal esketamine.

**Table 2:** Comparison of administration routes for esketamine and ketamine.

Route of administration	Bioavailability	Dosage
IV	100%	0.5–1.0 mg kg^−1^, twice weekly, 40–60 min each time, for 2 weeks
IM	90%‐95%	Possibly similar to intravenous injection
SC	90%‐95%	Possibly similar to intravenous injection
IN	30%‐50%	Esketamine: 56–84 mg, i.n., twice weekly, for 4 weeks Racemic ketamine: 50–150 mg, i.n., twice weekly
PO	10%‐20%	Significant changes (generally 2‐3 times per week, with 100–250 milligrams per dose being most acceptable)

IV, Intravenous; IM, Intramuscular; SC, Subcutaneous; IN, Intranasal; PO, Oral Administration.

TRD refers to depression that does not improve even after adequate and prolonged treatment with two or more different types of antidepressants that act through different mechanisms.^[^ ^196^ ^]^ The treatment duration for such patients is usually long, and they often need to try switching to different types of antidepressants, and traditional antidepressants take effect slowly. Recent studies have found that low‐dose ketamine infusion has a rapid and significant antidepressant effect.^[^ ^197^ ^]^ Based on this, esketamine nasal spray, as a modified form of ketamine, provides a more standardized, convenient, and risk‐controlled treatment option for patients with refractory depression. Therefore, we have summarized the overall efficacy of ketamine and esketamine in clinical trials for refractory depression in recent years (**Table** **3**).

**Table 3:** The effects and role of esketamine and ketamine in TRD.

Route of administration	Drug dosage	Trial plan	Follow‐up duration	Experimental results	Bias risk scores	References
Adolescent TRD	A single IV injection 0.5 mg kg^−1^ Ket within 40 min	Double‐blind, randomized, placebo‐controlled Con:Exp/8:8	within four weeks	Compared with midazolam, a single infusion of ket significantly reduced depressive symptoms 24 h after administration.	Low risk	[198]
Adults with severe depression	84 mg of nasal spray ESK	Double‐blind, randomized, placebo‐controlled Con:Exp/115:115	Lasted for four weeks	The ESK nasal spray can rapidly and significantly alleviate depressive symptoms in patients with severe depression who have a history of active suicidal intentions.	Low risk	[199]
Postpartum depression patients	A single IV 0.25 mg kg^−1^ ESK immediately after delivery.	Double‐blind, randomized, placebo‐controlled Con:Exp/150:148	Within 42 days after giving birth	In the perioperative period of elective cesarean section, IV administration of ESK can alleviate depressive symptoms in the early postpartum period.	Low risk	[200]
Patients with moderate to severe depression	Spray ESK 56mg / 84mg into the nose twice a week	Double‐blind, randomized, placebo‐controlled Con:Exp1:Exp2/113:117:116	Lasted for four weeks	Statistical significance was not achieved for the primary endpoint	Low risk	[201]
Prenatal depression patients	IV 0.2mg kg^−1^ ESK Within 40 min after giving birth	Double‐blind, randomized, placebo‐controlled Con:Exp/181:180	Postpartum, lasting for 42 days	For mothers with prenatal depression, a single low dose of ESK after childbirth decreases major depressive episodes at 42 days post partum by about three quarters.	Low risk	[202]
Patients with TRD	Received 6 doses of Ket within 3 weeks	Double‐blind, randomized, placebo‐controlled Con:Exp/170:195	Lasted for three weeks	For outpatients with moderate to severe non‐psychotic refractory depression, IV administration of ket shows a more significant antidepressant effect.	Some concerns	[203]
Patients with TRD	SC injections twice a week Ket 0.5–0.9 mg kg^−1^	Double‐blind, randomized, placebo‐controlled Con:Exp/54:54	Lasted for four weeks	Adequately dosed subcutaneous Ket was efficacious and safe in treating TRD over a 4‐week treatment period.	Low risk	[204]
Older patients with TRD	Flexible‐dose ESK nasal spray + new oral antidepressant drugs	Double‐blind, randomized, placebo‐controlled Con:Exp/66:72	Lasted for four weeks	ESK/antidepressant did not achieve statistical significance for the primary endpoint.	Some concerns	[205]

ESK,Esketamine; Ket,Ketamine; TRD,Treatment‐Resistant Depression; IV, Intravenous; SC, Subcutaneous; Con, Control group; Exp, Experimental group.

People with depression often have varying degrees of suicidal ideation (SI) and tendencies. The research has found that esketamine and ketamine can rapidly reduce patients' SI.^[^ ^206^ ^]^ Systematic reviews and meta‐analyses have also shown that single and multiple doses of ketamine/esketamine can produce an anti‐suicidal effect. Classic antidepressants take several weeks to months to produce maximum efficacy, whereas ketamine can produce antidepressant effects within hours, making it particularly suitable for the treatment of acute SI. Ketamine can also treat cross‐diagnostic aspects of SI, such as despair and anhedonia.^[^ ^4^ ^]^ Therefore, we summarized the overall effects of esketamine and ketamine on suicidal ideation in clinical trials in recent years (**Tables** **4** and **5**).

**Table 4:** The effects and role of ketamine on suicidal ideation.

Route of administration	Dose of ketamine	Trial plan	Follow‐up duration	Experimental results	Bias risk scores	References
Single IV infusion	0.2 mg kg^−1^	Double‐blind, randomized, placebo‐controlled Con:Exp/83:73	Within three days	Compared with placebo, BSS and MADRS‐SI↓after ketamine	Low risk	[207]
Single dose of IN ketamine	40 mg	Double‐blind, randomized, placebo‐controlled Con:Exp/15:15	Within 4 h after administration	Compared to placebo, MADRS‐SI↓ after ketamine	Low risk	[208]
Six IV infusions within 12 days	0.5 mg kg^−1^	Double‐blind, randomized, placebo‐controlled Con:Exp/4:5	Lasted for 42 days	SSI and MADRS‐SI ↓ compared to placebo	Some concerns	[209]
Single IV infusion	0.5 mg kg^−1^	Double‐blind, randomized, placebo‐controlled Con:Exp/40:40	Within 24 h after infusion	Compared with placebo, SSI ↓ after ketamine administration	Low risk	[210]
Two IV infusions, 24 h apart	0.5 mg kg^−1^	Double‐blind, randomized, placebo‐controlled Con:Exp/83:73	Within three days after administration	SSI↓ after ketamine Compared with placebo, depending on the diagnosis	Low risk	[211]
Three IV infusions within one week	0.5 mg kg^−1^	Single‐blind, randomized, placebo‐controlled Con:Exp/30:30	Last for a week	SSI ↓ after ketamine compared with placebo	High risk	[212]
Single IV infusion	0.5 mg kg^−1^	Single‐blind, randomized, placebo‐controlled Con:Exp/32:32	For a period of two consecutive months	SSI and MADRS‐SI ↓ compared to placebo	Low risk	[213]
Single IV infusion	0.1 mg kg^−1^, 0.5 mg kg^−1^ or 1.0 mg kg^−1^	Double‐blind, randomized, placebo‐controlled Con:Exp/16:40	Lasted for one month	The difference between ketamine and placebo in MADRS‐SI occurred on day 30 after infusion.	Low risk	[214]
Single IV infusion	0.5 mg kg^−1^	Double‐blind, randomized, placebo‐controlled Con:Exp/8:8	Within one day after administration	The numerical difference in SSI between ketamine and placebo may be related to improvements in memory and changes in serum brain‐derived neurotrophic factor.	Low risk	[215]
Single oral dose of ketamine	3mg kg^−1^	Double‐blind, randomized, placebo‐controlled Con:Exp/40:40	Within 7 days after administration	SSI score ↓ at 4 h, day 3, and day 7 after oral ketamine administration	Low risk	[216]
Single dose of IN ketamine	50mg	Single‐blind, randomized, placebo‐controlled Con:Exp/11:18	Lasting for 48 h	Compared with placebo, a statistically significant trend toward improvement in suicidal tendencies was observed in participants with AUD.	Low risk	[217]
Single IV infusion	0.5 mg kg^−1^	Double‐blind, randomized, placebo‐controlled Con:Exp/21:22	Within three days after administration	Compared with placebo, HDRS‐SI total score and CSSRS‐ISS total score ↓, improvement in brain network integrity	Not applicable	[218]

IV, Intravenous; IN, Intranasal; BSS, Beck Scale for Suicidal Ideation; MADRS‐SI, Suicide item of Montgomery‐Asberg Depression Rating Scale, SSI, Scale for Suicidal Ideation; AUD, Alcohol Use Disorder; HDRS‐SI, Suicide item of the Hamilton Depression Rating Scale; CSSRS‐ISS, Columbia‐Suicide Severity Rating Scale ‐ International Suicide Severity Item; Con, Control group; Exp, Experimental group.

**Table 5:** The effects and role of esketamine on suicidal ideation.

Route of administration	Dose of esketamine	Trial plan	Follow‐up duration	Experimental results	Bias risk scores	References
4 weeks, 8 times, IN esketamine	84mg	Single‐blind, randomized, placebo‐controlled Con:Exp/34:34	Lasted for two weeks	Compared with placebo, acute MADRS‐SI ↓ after ketamine; no difference in CGI‐S.	Low risk	[219]
4 weeks, 8 times, IN esketamine	84mg	Double‐blind, randomized, placebo‐controlled phase III Con:Exp/115:115	Lasting for 4 weeks	There was a numerical difference in CGI‐S between esketamine and placebo.	Low risk	[220]
Single IV injection of ketamine or IN injection of esketamine	0.25mg kg^−1^ esketamine, 0.5 mg kg^−1^ ketamine	Double‐blind, randomized, placebo‐controlled, Exp1: Exp2/30:29	Lasting for 7 days	The MADRS‐SI ↓ in the two treatment groups was comparable.	Low risk	[221]
IV injection of ketamine/IN injection of esketamine	differences	Cross‐over and parallel randomized clinical trials Not applicable	Lasting for 8 weeks	SI ↓ at acute time points, decreasing over time	Not applicable	[222]
Three IN infusions	0.25mg kg^−1^	Double‐blind, randomized, placebo‐controlled Con:Exp/27:27	Last for 5 days	Compared with placebo, C‐SSRS intention and intensity scores and MADRS‐SI ↓	Low risk	[223]
Twice weekly IN of esketamine	84mg	Double‐blind, randomized, placebo‐controlled Con:Exp/187:192	Lasted for 9 weeks	Median duration of remission and sustained remission ↓ in MDD	Low risk	[224]
Eight SC injections per week	The initial dose is 0.5 mg kg^−1^, which is adjusted continuously	open‐label Con:Exp/0:18	Lasted for 6 months	Compared with baseline scores, BSI scores and MADRS‐SI scores ↓	High risk	[225]

IV, Intravenous; SC, Subcutaneous; IN, Intranasal; MADRS‐SI, Suicide item of Montgomery‐Asberg Depression Rating Scale; CGI‐S, Clinical Global Impression‐Severity Scale; SI, Suicidal Ideation; C‐SSRS, Columbia‐Suicide Severity Rating Scale; MDD, Major Depressive Disorder; BSI, Brief Symptom Inventory; Con, Control group; Exp, Experimental group.

### 3.3 Potential Limitations

One potential problem faced by esketamine and ketamine is individual tolerance. A large meta‐analysis compared the tolerability of intravenous ketamine and intranasal esketamine in patients with major depressive disorder, finding that ketamine had higher overall response and remission rates than esketamine.^[^ ^222^ ^]^ Similarly, some patients may still experience temporary symptoms after receiving infusions of esketamine and ketamine, such as nausea, mental disassociation, dizziness, and unsteady gait.^[^ ^226^, ^227^, ^228^, ^229^ ^]^ Therefore, it is of utmost importance to ensure the safety of treatment for different individuals, especially for those patients who have varying absorption rates and lower tolerance levels.^[^ ^230^, ^231^ ^]^

Another issue that esketamine and ketamine face is the potential adverse reactions. Esketamine and ketamine may have varying degrees of effects on the cardiovascular system, urinary system, and nervous system. In the clinical trial of single‐dose esketamine treatment (56–84 mg nasal spray) approved by the FDA in 2025, the incidence of elevated blood pressure was ≈15% higher in the treatment group compared to the placebo group.^[^ ^227^ ^]^ For patients with uncontrolled hypertension, recent myocardial infarction, or unstable angina, it is still recommended to monitor blood pressure and electrocardiogram before. Among patients with treatment‐resistant depression who have been using intranasal esketamine for a long time (average exposure time of 42.9 months), ≈15.3% experience urinary tract infection symptoms (e.g., difficulty urinating, frequent urination, and urgency), but this is far lower than among recreational abusers (30–40%).^[^ ^232^ ^]^ Ketamine metabolites, such as demethylketamine, can directly damage bladder epithelial cells, inducing fibrosis and contractile dysfunction.^[^ ^233^, ^234^ ^]^ Therefore, it is clinically recommended to monitor urine routine (especially microalbumin) and bladder ultrasound during treatment, and encourage patients to drink ≥ 2 L of water daily to reduce drug concentration in the bladder. In addition, systematic assessment of cognitive function is necessary when using subanesthetic doses of ketamine for the treatment of depression in clinical practice.^[^ ^235^, ^236^ ^]^ Although some studies have suggested that intravenous administration of ketamine may cause transient fluctuations in attention and cognitive function within 24 h,^[^ ^237^, ^238^ ^]^ no evidence has yet been found that therapeutic doses of ketamine or esketamine cause persistent damage to cognitive function in patients with depression.^[^ ^239^, ^240^ ^]^ Therefore, when assessing the impact of ketamine or esketamine treatment on cognitive function in patients with depression, it is important to carefully distinguish between drug effects and cognitive impairment inherent to the disease, particularly when comparing treatment responders and non‐responders.

In terms of depression, ketamine can quickly exert an antidepressant effect, which is a cause for celebration. However, its efficacy is often relatively short‐lived, typically fading within one to two weeks after the treatment. Studies have shown that compared to a single administration, repeated ketamine infusions can to some extent prolong the duration of the antidepressant effect, and even maintain a certain efficacy after discontinuation. But another study found that the efficacy of twice‐weekly infusions is equivalent to that of three times‐weekly infusions, suggesting that an infusion frequency exceeding twice per week may not be necessary.^[^ ^241^, ^242^ ^]^ The long‐term use of ketamine may damage cognitive functions, particularly affecting attention, working memory, and episodic memory abilities.^[^ ^243^, ^244^, ^245^, ^246^, ^247^, ^248^, ^249^ ^]^ Studies have found that long‐term or high‐dose (5–20 milligrams per kilogram) exposure to ketamine may cause damage to the nervous system of rodents, but this dose is much higher than the conventional doses used in clinical treatment.^[^ ^246^ ^]^ Therefore, the frequency and dosage of ketamine use need to be further precisely determined.

## 4 Mechanism and Preclinical Application of Esketamine/Ketamine in Cancers

It has been demonstrated that NMDAR exhibits expression in a diverse range of cancer cells, encompassing neuronal tumors, thyroid cancer, lung cancer, colorectal cancer, breast cancer, T‐cell chronic lymphocytic leukemia, multiple myeloma, laryngeal cancer, pancreatic cancer cells, and prostate cancer, among others.^[^ ^31^, ^250^, ^251^ ^]^ Moreover, the expression level of NMDAR increased with the increase of tumor malignancy, suggesting that abnormal expression of NMDAR may have a potential carcinogenic effect.

### 4.1 Mechanism of Esketamine/Ketamine in Cancers

At present, the underlying mechanism of the abnormal expression of NMDAR in carcinogenesis is not yet fully understood. The activation of these synapses between cancer cells and neurons has been found to be associated with the release of glutamate from these synapses, the growth of tumors, and the migration of cancer cells.^[^ ^252^ ^]^ The synaptic characteristics mediated by neurotransmitter receptors (e.g., NMDAR) have been observed not only in glioblastoma but also in invasive breast cancer with brain metastasis.^[^ ^252^, ^253^ ^]^ Calcium signaling pathways generated in NMDAR and tumor cells are activated by the synaptic release of glutamate. Glutamate appears to be secreted primarily through the xCT‐glutamate/cystine reverse transporter protein: one glutamate is exported, one cystine is imported, and rapidly converted intracellularly to cysteine for the production of GSH.^[^ ^254^ ^]^ This contributes to an increase in the intracellular concentration of calcium ions. The increase in calcium ion concentration has been demonstrated to activate signaling pathways such as CaMKII and MAPK, thereby promoting the activation of CREB (cAMP response element‐binding protein) in tumor cells.^[^ ^255^, ^256^ ^]^ In the context of glioblastoma, the CREB protein has been observed to induce the expression of immediate early genes, such as cFos, by stimulating the activity of Top2β, also known as topoisomerase IIβ. This induction results in the formation of DNA double‐strand breaks (DSBs) within the promoter region of the target genes.^[^ ^257^ ^]^ In neurons, DSBs have been shown to promote rapid transcription of early response genes.^[^ ^258^ ^]^ In contrast, in tumor cells, DSBs have been demonstrated to result in genome rearrangement, a hallmark of cancer.^[^ ^259^ ^]^

At present, the research on the signaling of NMDAR dysfunction mainly focuses on the field of neuroscience. For instance, genetic studies have demonstrated that GKAP (also known as DLGAP1), a core backbone protein of NMDAR, may influence the progression of pancreatic neuroendocrine cancer by regulating downstream effector molecules such as HSF1 (heat shock transcription factor 1) and FMRP (fragile X intelligence blocking protein).^[^ ^260^ ^]^ Moreover, transcriptomic signatures indicative of low activity or inhibition of the NMDAR‐DLGAP1 pathway have been demonstrated to be associated with improved outcomes in patients diagnosed with various cancers, including pancreatic, brain, kidney, and uveal melanoma.^[^ ^260^ ^]^ Subsequent studies have identified that high expression of DLGAP1 is also associated with more aggressive subtypes of breast cancer.^[^ ^252^ ^]^ However, no current study has directly suggested that the NMDAR‐DLGAP1 pathway is directly related to the antitumor effects of ketamine and esketamine. Given the established role of DLGAP1 in NMDAR signaling, it can be hypothesized that it may be indirectly implicated in the regulation of the anti‐tumor mechanism of ketamine and esketamine. Consequently, further research is required to substantiate whether DLGAP1 is indeed involved in the anti‐tumor effects of ketamine and esketamine, and to investigate the potential value of its utilisation in anti‐tumor therapy.

**Figure 5:**
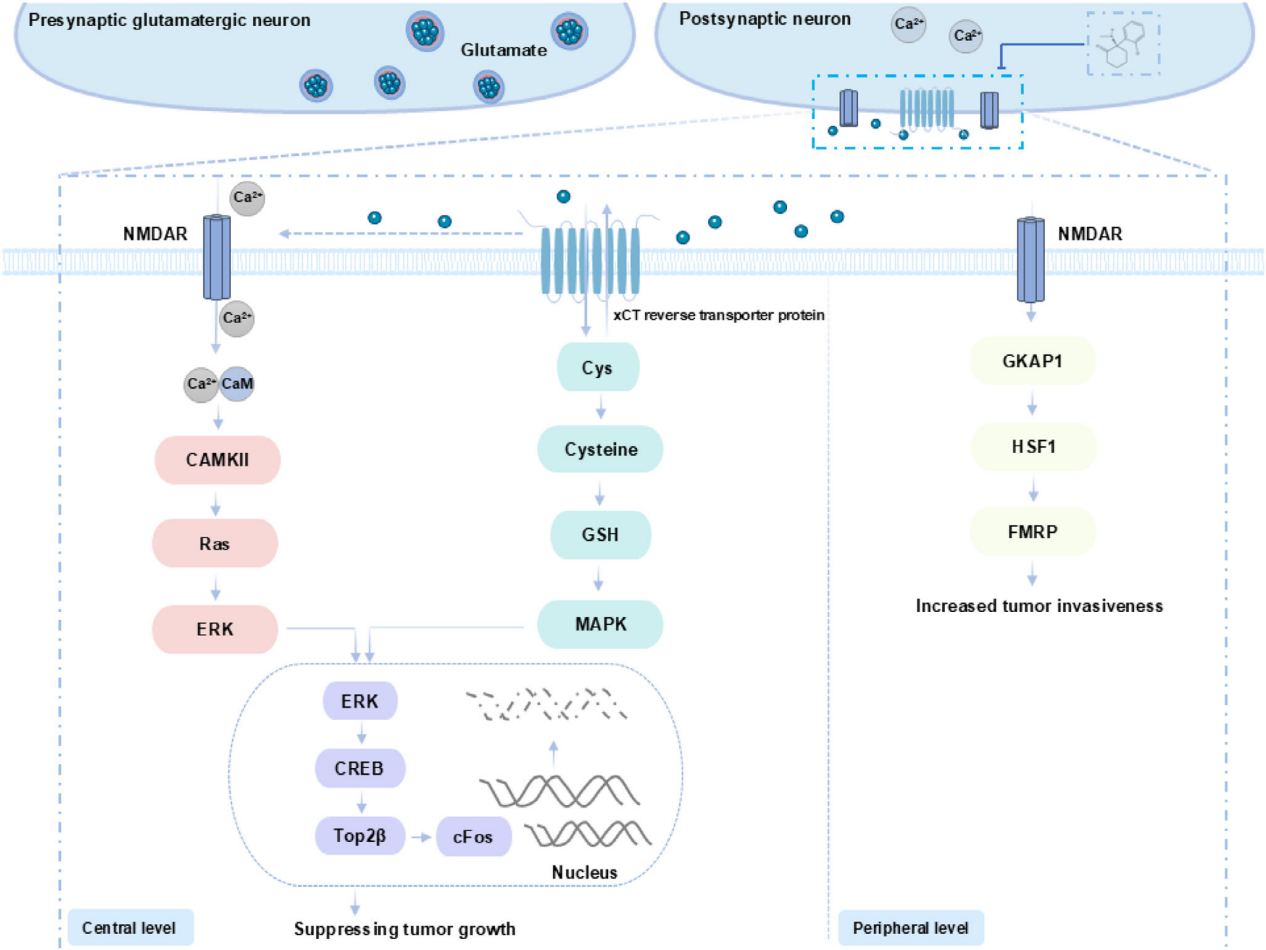
Role of Esketamine/ketamine in cancers. In typical circumstances, glutamate released from presynaptic neurons enters the synaptic gap. Acting on NMDAR, glutamate increases the calcium ion concentration in postsynaptic neurons, thus activating CaMKII and MAPK signalling pathways and further promoting the activation of CREB protein in tumor cells. The subsequent expression of cFos is then induced by CREB protein, through the stimulation of Top2β activity, which in turn results in the formation of DNA double strand breaks in the promoter region of the target gene. In addition, glutamic acid can be exported from the cell by the XCT‐glutamic acid/cystine reverse transporter. Cystine, when added to the outside of the cell, can be reduced to cysteine, which can then be converted into GSH and, in turn, affect the MAPK pathway. Ketamine and esketamine inhibit this pathway by binding to NMDAR. Second, the study also suggests that the core skeleton protein GKAP1 of NMDAR may influence tumor progression by regulating downstream effector molecules such as HSF1 and FMRP. However, it remains to be demonstrated whether esketamine or ketamine act on this pathway.

NMDAR subtypes are also closely related to the development of tumors. It is composed of two essential GluN1 subunits, along with two additional subunits that can be either GluN2 or GluN3.^[^ ^261^ ^]^ The Studies have shown that silencing of the GRIN2A inhibits the proliferation of MKN45 gastric cancer cells and promotes cell cycle arrest.^[^ ^262^ ^]^ Knocking down the GRIN1 will reduce the cell viability of rhabdomyosarcoma and medulloblastoma (TE671) cells.^[^ ^250^ ^]^ Knocking down the GRIN2B reduced cell proliferation in the co‐culture of malignant breast cancer cells and neurons.^[^ ^252^ ^]^ The research also found that NMDAR antagonists (e.g., MK‐801, ifenprodil, AP5, and memantine) can inhibit the proliferation of cancer cells, alter the morphology and motility of cancer cells, and affect the growth and progression of tumors. Although current basic research has suggested that ketamine and esketamine can exert anti‐tumor effects by inhibiting NMDAR, they are still broad‐spectrum NMDAR antagonists (with the same inhibitory effect on NMDAR in healthy neurons). However, the research has not specifically delved into the specific subtypes of NMDAR affected by ketamine and esketamine, nor has it provided detailed mechanisms (**Figure** **5**).

### 4.2 Preclinical Application of Esketamine/Ketamine in Cancers

The clinical application of esketamine and ketamine in anti‐tumor treatment remains unexplored. Currently, the anti‐tumor effects of esketamine mainly focus on the biological behavior of cancer cells and the dysfunction of NMDAR. In vitro study demonstrated that esketamine effectively suppressed the proliferation, migration, and invasion of two esophageal squamous cell carcinoma (ESCC) cell lines in a dose‐ and time‐dependent manner, while also promoting apoptosis. Furthermore, esketamine significantly down‐regulated the expression of ERCC6L, AHR, and KIF2C proteins in ESCC cells, indicating its potential as an anti‐ESCC agent in vitro.^[^ ^263^ ^]^ Another study revealed that esketamine inhibited the proliferation and migration of nasopharyngeal carcinoma (NPC) cells and induced apoptosis through the PERK/ATF4/CHOP signaling pathway mediated by NMDAR. Additionally, when combined with cisplatin, esketamine enhanced cisplatin's inhibitory effect on NPC cell proliferation.^[^ ^264^ ^]^ These findings provide novel insights into the application of esketamine for cancer treatment and suggest a potential clinical approach combining it with cisplatin for nasopharyngeal cancer treatment. In pancreatic cancer research, it was observed that pancreatic cancer cells expressed GluN2A. Continuous stimulation with 5 µM ketamine or esketamine for 24 h or 48 h significantly inhibited PaTu8988t pancreatic cancer cell proliferation while reducing nuclear NFATc2 expression (a specific NFAT protein).^[^ ^31^ ^]^ However, after 72 h of continuous stimulation, NFATc2 expression was notably increased, suggesting potential adverse effects from long‐term exposure to these substances.^[^ ^264^ ^]^ Conflicting results suggest that prolonged exposure may trigger a potential risk of adaptive tumor response, rebounding line proliferation, or drug toxicants.

Currently, ketamine has also been extensively studied in various types of tumor cells, and it has been found that ketamine has varying degrees of inhibitory effects on breast cancer,^[^ ^265^, ^266^ ^]^ colorectal cancer,^[^ ^267^, ^268^ ^]^ liver cancer,^[^ ^269^ ^]^ lung cancer,^[^ ^270^ ^]^ and pancreatic cancer,^[^ ^31^, ^264^ ^]^ etc. Aerobic glycolysis plays a crucial role in cancer progression. Research has found that ketamine can inhibit the aerobic glycolysis of colorectal cancer cells by suppressing the NMDAR‐CaMK II‐c‐Myc pathway, thereby weakening the vitality and migration of colon cancer cells.^[^ ^268^ ^]^ In the field of liver cancer, studies have found that ketamine can induce ferroptosis in liver cancer cells by targeting lncRNA PVT1/miR‐214‐3p/GPX4,^[^ ^269^ ^]^ or it can inhibit proliferation and induce ferroptosis and cell apoptosis in breast cancer cells by targeting the KAT5/GPX4 axis.^[^ ^265^ ^]^ In 2018, Zhou and his colleagues discovered that the activation markers CD69 of white blood cells and natural killer cells were downregulated in human lung adenocarcinoma tissue samples. Ketamine upregulated CD69, resulting in an increase in apoptosis of cancer cells.^[^ ^270^ ^]^

### 4.3 Potential Limitations

According to the existing studies, esketamine and ketamine have potential research value in anti‐tumor effect. As previously mentioned, esketamine has different degrees of tumor inhibition in esophageal squamous cell carcinoma, nasopharyngeal carcinoma, and pancreatic cancer. However, the anti‐tumor research on esketamine and ketamine is currently limited to the exploratory stage of preclinical and basic experimental studies. Preliminary studies have shown that esketamine and ketamine have potential anti‐tumor effect, but there is still a lack of large‐scale animal experiments, clinical trials, and reliable biomarkers to verify. In addition, some studies have also found that ketamine has an opposite effect on tumors. One study discovered that ketamine addiction upregulates the expression of genes involved in the Wnt, EGFR, and BMP signaling pathways, which may be related to the progression and metastasis of breast cancer.^[^ ^271^ ^]^ In another breast cancer study, it was found that ketamine may promote tumor growth through miR‐27b‐3p, HER2, and EGFR in the tumor microenvironment.^[^ ^266^ ^]^ Therefore, in future cancer research, it is necessary to further clarify the exact molecular mechanisms by which ketamine and esketamine promote and inhibit cancer, in order to avoid related risks. In addition, it is important to pay attention to the time‐dependent properties of ketamine and esketamine. They may inhibit tumor growth in the short term, but the long‐term effects remain unknown.

## 5 Mechanisms and Clinical Applications of Esketamine/Ketamine in Immune Inflammation: From Brain, Heart, Liver to Lung

Immunoinflammation is a fundamental defence mechanism of the body against tissue infection or pathological damage. This process involves the regulation of immune cells, the release of inflammatory mediators, and the coordinated action of multiple signaling pathways, with the overall goal of removing pathogens from the body and restoring homeostasis to the internal environment. However, disturbances in the regulatory mechanisms of immunoinflammation may trigger chronic inflammatory responses that can lead to a range of disease states, such as rheumatoid arthritis, inflammatory bowel disease, and neurodegenerative disorders. Current research has found that ketamine and esketamine exert specific immunomodulatory and anti‐inflammatory effects on organ injuries such as those to the brain, heart, liver, and lungs.

### 5.1 Mechanisms of Esketamine/Ketamine in Immune Inflammation: From Brain, Heart, Liver to Lung

#### 5.1.1 Mechanism of Esketamine/Ketamine in Immune Inflammation in the Brain

Ketamine and esketamine play a role in perioperative cognitive impairment, Alzheimer's disease, and cerebral brain protection. Perioperative neurocognitive disorder (PND) is a common complication among elderly patients after surgery. Research has shown that ketamine and esketamine can alleviate postoperative cognitive dysfunction in mice by inhibiting M1 polarization and inflammatory responses of microglia, regulating the BDNF‐TrkB pathway, and stimulating the interferon gene.^[^ ^272^, ^273^ ^]^ Alzheimer's disease (AD) is a neurodegenerative disorder characterized by the accumulation of beta‐amyloid protein (Aβ) and the subsequent inflammatory response, which ultimately leads to neuronal damage.^[^ ^274^ ^]^ Esketamine has been found to inhibit neuroinflammation through the PI3K/AKT pathway, thereby significantly alleviating the cognitive deficits and impaired memory function in 3xTg‐AD mice.^[^ ^275^ ^]^ In terms of cerebral brain protection (CBP), reactive oxygen species (ROS) interact with substances such as brain‐derived neurotrophic factor ‐ tyrosine kinase receptor B (BDNF‐TrkB), STING/TBK1, etc. The accumulation of ROS may affect the survival and apoptosis of nerve cells.^[^ ^276^ ^]^ However, in the STING/TBK1 pathway, eslicarbazepine acetate may exert a protective effect by inhibiting the STING signaling pathway and preventing the initiation of immune responses such as interferon.^[^ ^277^ ^]^

#### 5.1.2 Mechanism of Esketamine/Ketamine in Immune Inflammation in the Heart

Ketamine and esketamine exert anti‐inflammatory and cardioprotective effects through multiple target actions. They can regulate the immune imbalance during surgery or stress conditions by inhibiting the production of pro‐inflammatory cytokines and stimulating the release of anti‐inflammatory mediators. They can also enhance the cellular defense mechanism by activating endogenous antioxidant pathways such as AMPK/Nrf2/HO‐1 and heme oxygenase system.^[^ ^278^, ^279^, ^280^ ^]^

Esketamine can also regulate the expression of TRPV1 in the transient receptor potential (TRP) family, which can inhibit intracellular calcium ion concentration and alleviate myocardial cell damage caused by hypoxia/reoxygenation.^[^ ^281^ ^]^ These mechanisms work together, ultimately resulting in a decrease in the peak levels of myocardial injury markers and a reduction in the severity of myocardial injury. The key underlying mechanisms are summarized in **Table** **6**.

**Table 6:** The protective mechanism of esketamine in cardiac injury.

Protective Mechanism	Description
Electrophysiology	Ketamine reduces the peak current of sodium ions, esketamine has no effect on the behavior of sodium ion channels^[^ ^282^ ^]^ Esketamine inhibits intracellular Ca^2+^ concentration^[^ ^281^ ^]^
Enhance endogenous antioxidant protection mechanisms	Activate the Nrf2/HO‐1 signaling pathway^[^ ^278^ ^]^ AMPK/Nrf2 signaling pathway^[^ ^279^ ^]^ AMPK‐mTOR signaling pathway^[^ ^280^ ^]^
Inhibition of cardiomyocyte apoptosis	PI3K/Akt signaling pathway^[^ ^283^ ^]^ Regulating TRPV1 expression^[^ ^281^ ^]^ Alleviate cardiomyocyte ferroptosis via STING^[^ ^284^ ^]^

Nrf2,Nuclear factor erythroid 2‐related factor 2; HO‐1,Heme oxygenase‐1; TRPV1, Transient Receptor Potential Vanilloid 1.

#### 5.1.3 Mechanism of Esketamine/Ketamine in Immune Inflammation in the Liver

The main problem encountered during liver surgery is ischemia‐reperfusion injury (I/R injury) in Liver Transplant patients. Current research has found that this injury involves multiple mechanisms. In the early stage of its dynamic pathological process (within 6 h of reperfusion), DAMPs (e.g., HMGB1) are mainly released,^[^ ^285^ ^]^ and Kupffer cellsv^[^ ^286^ ^]^ are activated through TLR4/NF‐κB pathway to release inflammatory factors such as TNF‐α and IL‐6.^[^ ^287^, ^288^, ^289^ ^]^ Then the early inflammatory storm transitioned to oxidative stress^[^ ^290^ ^]^ leading to iron death^[^ ^291^ ^]^ and Nrf2 compensatory activation,^[^ ^292^ ^]^ and the late inflammatory‐oxidative vicious cycle formed. Li et al conducted a study utilizing a rat model of autologous liver transplantation, where they observed that ketamine markedly suppressed the expression of inflammatory cytokines such as IL‐6, TNF‐α, IL‐1, and IL‐10. Furthermore, they demonstrated that ketamine inhibited the proinflammatory cascade initiated by Kupffer cells (KCs) via the NF‐κB signaling pathway, thereby enhancing the resolution of aseptic inflammation and mitigating liver ischemia reperfusion injury.^[^ ^293^ ^]^ Gundogdu et al further explored the effect of ketamine by administering two distinct doses (high and low), revealing a dose‐dependent anti‐inflammatory action of ketamine in the context of liver I/R injury.^[^ ^294^ ^]^

#### 5.1.4 Mechanism of Esketamine/Ketamine in Immune Inflammation in the Lung

Acute respiratory distress syndrome (ARDS) is an acute inflammatory disease.^[^ ^295^ ^]^ This disease is typically characterized by extensive inflammatory infiltration and degeneration of alveolar epithelial cells and endothelial cells, resulting in increased vascular permeability and pulmonary edema.^[^ ^296^ ^]^ The research has found that esketamine can protect the lungs by inhibiting ferroptosis of cells, reducing pulmonary inflammation, and regulating the HIF‐1α/HO‐1 pathway.^[^ ^297^ ^]^ Xie et al have reported that esketamine acts by down‐regulating the activation of Wnt/β‐catenin signaling in the lung and inhibiting the migration of small intestinal s IL‐17‐producing inflammatory γδT cells (γδT17 cells) into the lung.^[^ ^298^ ^]^ Esketamine's lung‐protective properties also stem from its ability to inhibit the MAPK/NF‐κB signaling pathway and oxidative stress. Acute lung injury is a systemic inflammatory response caused by the imbalance of anti‐inflammatory and pro‐inflammatory cytokines, and it is often accompanied by the production of oxygen free radicals.^[^ ^96^ ^]^ Ketamine and esketamine can directly inhibit the production of pro‐inflammatory factors, reduce the degree of postoperative systemic inflammatory response of patients, and reduce the level of tissue oxidative stress by enhancing the expression activity of Superoxide dismutase (SOD), further reduce the generation of free radicals and enhance the antioxidant capacity of the body.^[^ ^299^ ^]^ In experimental settings, mechanical ventilation in COPD mice prompted a surge in cytokine levels, provoking alveolar inflammation and culminating in acute lung injury. Esketamine, by targeting the MAPK/NF‐κB signaling pathway, mitigates inflammation and oxidative stress, thereby improving lung injury.^[^ ^299^ ^]^

### 5.2 Clinical Applications of Esketamine/Ketamine in Immune Inflammation: From Brain, Heart, Liver to Lung

#### 5.2.1 Clinical Application of Esketamine/Ketamine in Immune Inflammation in the Brain

Current clinical evidence supports that sub‐anesthetic doses of ketamine and esketamine are promising perioperative intervention measures for high‐risk patients (e.g., derly people undergoing major surgeries), as they provide an important mechanism for preventing related brain disorders through systemic and central anti‐inflammatory and immunomodulatory effects, in addition to traditional anesthesia and analgesia.^[^ ^300^ ^]^ Randomized controlled trials have shown that the application of low‐dose esketamine in elderly gastrointestinal tumor surgery can effectively reduce the levels of postoperative pro‐inflammatory cytokines (e.g., IL‐6), and help alleviate the immunosuppression caused by surgical stress, which is related to the improvement of postoperative cognitive function in patients.^[^ ^301^, ^302^ ^]^ Multiple systematic reviews and clinical trial results have shown that perioperative adjunctive use of esketamine is associated with a reduced incidence of postoperative delirium and improved early neurocognitive recovery indicators. These positive neurobehavioral outcomes are believed to be driven by its effective control of perioperative abnormal inflammatory and immune levels.^[^ ^303^, ^304^, ^305^, ^306^ ^]^

#### 5.2.2 Clinical Application of Esketamine/Ketamine in Immune Inflammation in the Heart

Ketamine and esketamine have demonstrated anti‐inflammatory effects and potential cardiac protective values in cardiac surgeries. In non‐extracorporeal circulation Coronary Artery Bypass Grafting (CABG), low‐dose ketamine can effectively inhibit the release of pro‐inflammatory factors and significantly reduce the levels of inflammatory markers such as IL‐6 and TNF‐α after the operation.^[^ ^307^ ^]^ During the extracorporeal circulation CABG surgery, continuous infusion of esketamine can effectively weaken the pro‐inflammatory cytokine response triggered by cardiopulmonary bypass.^[^ ^308^ ^]^ Further studies comparing the results found that the anesthetic regimen based on ketamine and dexmedetomidine demonstrated superior cardiopreconditioning effects in cardiac bypass surgery compared to the fentanyl and propofol regimen, with less release of postoperative myocardial injury biomarkers.^[^ ^309^ ^]^ Observational studies have confirmed that compared with sevoflurane‐sufentanil anesthesia, patients undergoing cardiac surgery under ketamine‐dexmedetomidine anesthesia show more favorable trends in cardiac biomarker changes.^[^ ^310^ ^]^ These pieces of evidence collectively indicate that ketamine‐based drugs exert an important Myocardial Protective (MCP) effect during the perioperative period of cardiac surgery by regulating the inflammatory immune response.

#### 5.2.3 Clinical Application of Esketamine/Ketamine in Immune Inflammation in the Liver

Currently, the clinical studies on the anti‐inflammatory and anti‐immune effects of ketamine and esketamine in liver surgeries have yielded inconsistent results. The effectiveness may highly depend on the specific type of surgery and the intensity of inflammatory stress. In liver resection surgeries, the research results are divergent: a randomized double‐blind trial for liver resection patients requiring the Pringle maneuver clearly showed that ketamine failed to effectively inhibit the synthesis of IL‐6.^[^ ^311^ ^]^ However, another randomized double‐blind study on liver resection found that the combined infusion of dexmedetomidine and ketamine had a regulatory effect on the inflammatory response.^[^ ^312^ ^]^ In studies of non‐direct liver‐related surgeries, such as microvascular reconstruction in the head and neck region that focus on liver function, sub‐anesthetic doses of esketamine show good postoperative liver function safety.^[^ ^313^ ^]^ These pieces of evidence collectively suggest that the liver anti‐inflammatory effect of ketamine and esketamine may be significantly influenced by the surgical background and the degree of immune activation. The potential value of these drugs in major surgeries such as liver transplantation merits further exploration.

#### 5.2.4 Clinical Application of Esketamine/Ketamine in Immune Inflammation in the Lung

Ketamine and esketamine are closely related to anti‐inflammatory and immunomodulatory effects in lung surgeries and acute lung injuries. Zhao et al implemented esketamine in the radical resection of lung cancer and observed significant reductions in pro‐inflammatory factors, including TNF‐α and IL‐6, alongside diminished oxidative stress, enhanced bronchodilation, and lowered airway resistance, ultimately mitigating lung injury.^[^ ^314^ ^]^ Ketamine and esketamine can improve oxygenation in ARDS patients,^[^ ^315^ ^]^ and there are meta‐analyses supporting its reduction in postoperative pulmonary complications.^[^ ^316^ ^]^ Ketamine can also effectively alleviate the infiltration of inflammatory cells and the release of pro‐inflammatory cytokines in the acute lung injury model caused by mechanical ventilation.^[^ ^317^ ^]^ In conclusion, the existing evidence strongly indicates that ketamine and esketamine, through their anti‐inflammatory properties, can exert lung‐protective functions in different clinical scenarios.

### 5.3 Potential Limitations

From the existing research, it is obvious that ketamine and esketamine have certain anti‐inflammatory and anti‐immune properties. However, the immunomodulatory effects of ketamine and esketamine during use may be closely related to the dosage.^[^ ^318^, ^319^, ^320^ ^]^ Cho et al. found in their experiments that in patients undergoing colorectal cancer surgery, the intraoperative use of low‐dose ketamine had no favorable effect on the overall activity of natural killer cells, inflammatory response, or prognosis after the operation. This phenomenon may be partly attributed to the relatively low degree of postoperative inflammation activation during laparoscopic surgery and the use of a low dose of ketamine,^[^ ^321^ ^]^ but its authenticity still requires further verification. In another experiment, ketamine might promote the release of inflammatory factors by activating the TLR4 signaling pathway or inducing oxidative stress.^[^ ^318^ ^]^ Correctly understanding the dual nature of these two drugs in immune regulation is crucial. Further research is needed in the future to clarify the optimal dosage, administration methods, drug combination evaluation, and the mechanism of action of ketamine and esketamine in different treatment outcomes. This may provide new targets and strategies for the clinical application of ketamine and esketamine.

## 6 Conclusion 

Esketamine and ketamine, as NMDAR antagonists, exhibit multi‐modal effects in clinical applications. In perioperative management, they exert analgesic effects by blocking NMDAR, regulating the opioid system, and inhibiting the central sensitization process. Their regulation of HCN channels endows them with good sedative properties. These drugs can maintain hemodynamic stability during anesthesia, alleviate respiratory depression, and are particularly suitable for short surgeries, regional anesthesia assistance, and special populations (e.g., pediatric, obstetric, and elderly patients).

In the field of mental illness treatment, esketamine and ketamine exert rapid antidepressant effects by regulating the glutamatergic system and non‐glutamatergic systems (including monoamine neurotransmitters, opioid receptors, etc.). Studies have shown that these drugs can significantly improve the symptoms of patients with refractory depression within a few hours, especially for those with suicidal risk, which has important therapeutic value. The mechanism of action involves multiple aspects, including promoting the release of BDNF, activating the mTOR signaling pathway, and regulating synaptic plasticity, etc.

**Figure 6:**
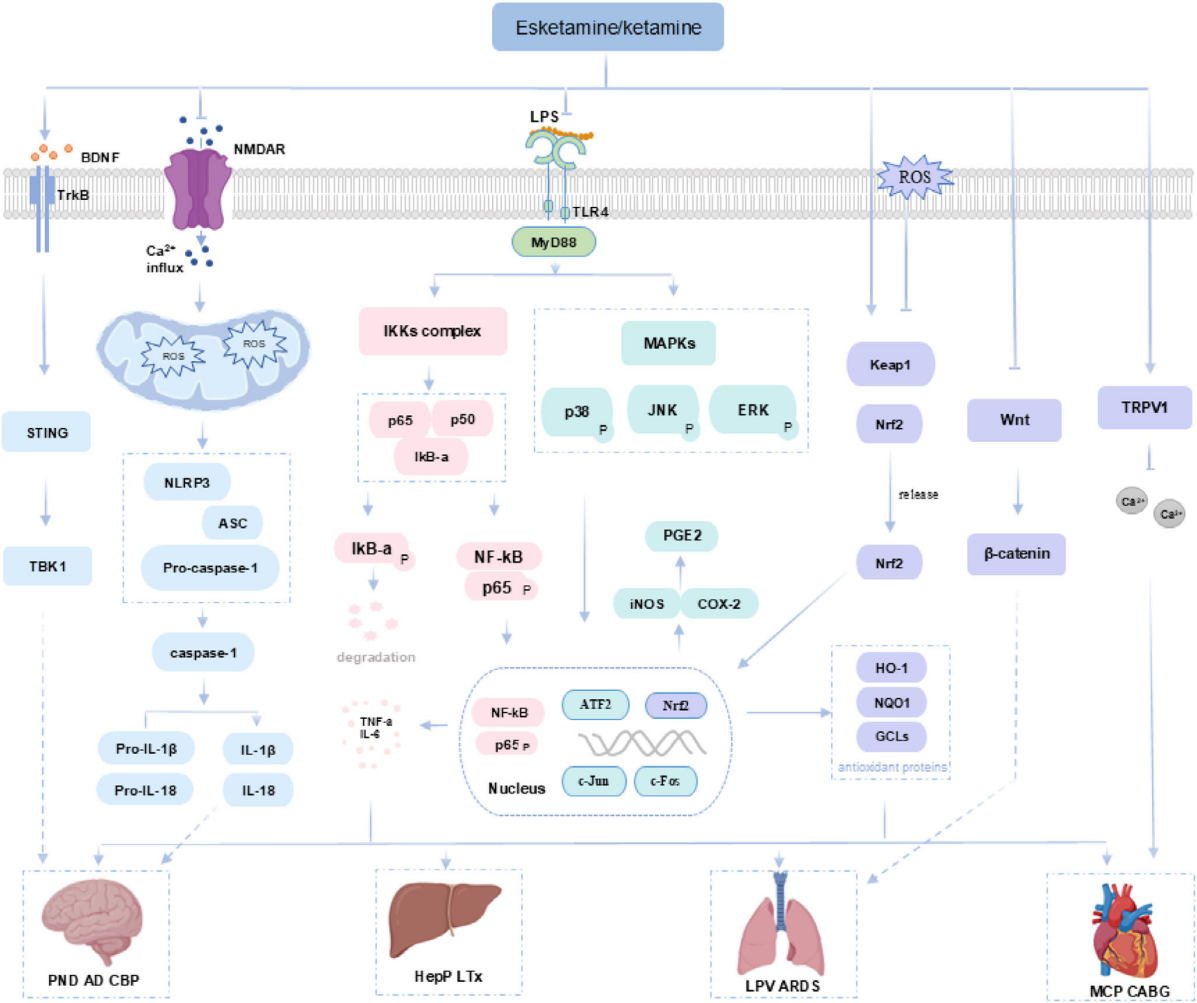
Role of Esketamine/ketamine in immune inflammation. In multiple organ systems such as the brain, heart, liver, and lungs, ketamine and esketamine exert protective effects through anti‐inflammatory, antioxidant, and regulation of specific signaling pathways (e.g., inhibition of NF‐κB and activation of Nrf2/HO‐1, etc.). Clinical studies have shown that they can alleviate perioperative neurocognitive disorders, inhibit the release of myocardial injury markers, regulate liver inflammatory responses, and reduce the inflammation and oxidative stress of acute lung injury. They particularly show potential application value in high‐risk situations such as cardiac surgery, elderly patients, and acute respiratory distress syndrome.

Studies have shown that the anti‐tumor effects of esketamine and ketamine are exerted through the inhibition of NMDAR. The mechanism may involve calcium influx triggered by glutamate‐mediated activation of NMDAR and the downstream CaMKII/MAPK‐CREB signaling pathway, thereby regulating the expression of tumor‐related genes. Although genomics suggests that the NMDAR‐DLGAP1 signaling pathway is related to tumor development, the association between ketamine and this pathway is still unclear. Currently, related research is still limited to basic tumor biology and lacks systematic preclinical evidence to support its clinical application (**Figure** **6**).

**Figure 7:**
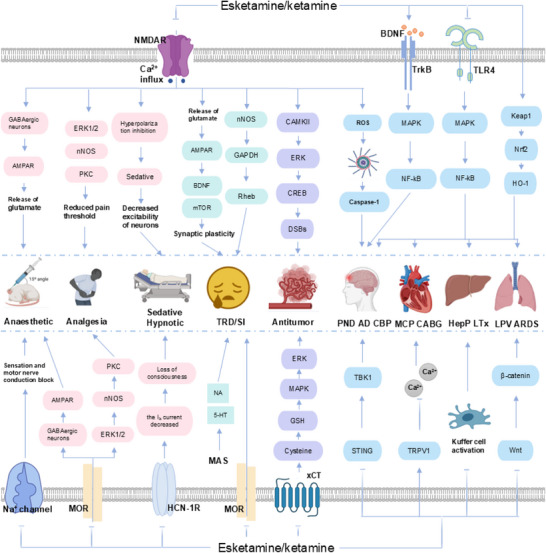
Summary of the effects of Esketamine/ketamine. Esketamine is the right‐handed isomer of ketamine. Based on the clinical application effects of esketamine and ketamine, this figure mainly summarizes from four aspects: classic applications (analgesia, sedation, and anesthesia), antidepressant effects, anti‐tumor effects, as well as the related mechanisms and clinical applications of anti‐inflammatory and immune‐inflammatory effects.

In multiple organ systems such as the brain, heart, liver, and lungs, esketamine and ketamine exert protective effects through anti‐inflammatory, antioxidant, and regulation of specific signaling pathways (e.g., inhibition of NF‐κB and activation of Nrf2/HO‐1, etc.). Clinical studies have shown that they can alleviate perioperative neurocognitive disorders, inhibit the release of myocardial injury markers, regulate liver inflammatory responses, and reduce the inflammation and oxidative stress of acute lung injury. They particularly show potential application value in high‐risk situations such as cardiac surgery, elderly patients, and acute respiratory distress syndrome (**Figure** **7**).

It is important to note that although short‐term use under medical supervision is relatively safe, long‐term or high‐dose use may lead to adverse reactions, including urinary system toxicity (e.g., interstitial cystitis), cognitive dysfunction, addiction tendencies, and mental symptoms, etc. Therefore, in clinical applications, the risk‐benefit ratio should be strictly evaluated, and the use should be under professional medical supervision.

## 7 Future Perspectives

Esketamine and ketamine still have some issues to be resolved, and in future developments, tolerance and psychiatric side effects, such as dissociative symptoms may occur with prolonged use. Safety and the optimal dosage are also key issues surrounding the use of the drug. Future research can explore this issue by differentiating the mode of administration (e.g., long‐acting formulations or targeted delivery techniques), classifying special populations (e.g., children, the elderly, and critically ill patients), and clarifying the stage of use (e.g., on‐site anesthesia and obstetric analgesia).

In the field of antidepressant therapy, while focusing on the traditional NMDAR antagonism mechanism, esketamine and ketamine should further explore new therapeutic targets. Future directions could be in focusing on the role of esketamine in neuroplasticity, epigenetic regulation, and neuroinflammation, exploring its safety in different age groups, and developing novel derivatives with fewer side effects. The exploration of low‐dose combination therapies, such as combining with anti‐inflammatory or neuroprotective agents, holds promise in optimizing the treatment of depression and reducing side effects.

In the field of tumor suppression, esketamine and ketamine are in the future research pathway to play a role in inhibiting tumor growth and metastasis with NMDAR antagonism, immunomodulation, and anti‐inflammation. In addition, the analgesic and antitumor effects of esketamine can be combined with immunotherapy (e.g., PD‐1/PD‐L1 inhibitors), targeted therapies, and radiation therapy to improve patient survival. Future studies may experiment with innovative low‐concentration delivery systems, such as nanoparticles, for the treatment of refractory tumors, including glioblastoma, triple‐negative breast cancer, and metastatic tumors.

In the field of immuno‐inflammation, esketamine and ketamine are equally promising. In terms of anti‐inflammatory mechanisms, esketamine could focus on modulating the gut microbiota‐brain axis, repairing mitochondrial function, and novel cell death pathways (e.g., cellular pyrolysis and iron death). In addition, targeting chronic inflammatory conditions such as metabolic syndrome and aging‐associated inflammation, esketamine could open up new avenues for the prevention and treatment of chronic diseases. The emergence of new technologies and multidisciplinary collaboration can further facilitate the translation of basic research to clinical applications in the field of immunoinflammation and provide better solutions for related diseases.

## Conflict of Interest

The authors declare no conflict of interest.

## Author Contributions

Y.W., J.X., and X.Z. contributed equally to the work. Y.W., Z.Y., X.Z., M.G., and Y.L. were responsible for methodology, investigation, and writing the original draft. Y.W., Z.Y., X.Z., Y.W., J.H., and L.C. contributed to methodology, investigation, and validation. L.C., J.H., and Y.W. contributed to the investigation and validation. J.X., J.H., and Y.W. were responsible for validation and formal analysis resources, and data curation. Z.Y., M.G., Y.L., and J.X. were involved in conceptualization, resources, writing, reviewing, editing, supervision, funding acquisition, and project administration.
